# Major depression symptom severity associations with willingness to exert effort and patch foraging strategy

**DOI:** 10.1017/S0033291724002691

**Published:** 2024-11

**Authors:** Laura A. Bustamante, Deanna M. Barch, Johanne Solis, Temitope Oshinowo, Ivan Grahek, Anna B. Konova, Nathaniel D. Daw, Jonathan D. Cohen

**Affiliations:** 1 Princeton Neuroscience Institute, Princeton University, Princeton, NJ, USA; 2 Department of Psychological & Brain Science, Washington University in St. Louis, St. Louis, MO, USA; 3 Department of Psychological & Brain Science and Psychiatry, Washington University in St. Louis, St. Louis, MO, USA; 4 Department of Psychiatry, Rutgers University, New Brunswick, NJ, USA; 5 Princeton Neuroscience Institute and Department of Molecular Biology, Princeton University, Princeton, NJ, USA; 6 Department of Cognitive and Psychological Sciences, Brown University, Providence, RI, USA; 7 Princeton Neuroscience Institute and Department of Psychology, Princeton University, Princeton, NJ, USA

**Keywords:** computational psychiatry, major depressive disorder, effort-based decision-making, cognitive control, physical effort, foraging, anxiety

## Abstract

**Background:**

Individuals with major depressive disorder (MDD) can experience reduced motivation and cognitive function, leading to challenges with goal-directed behavior. When selecting goals, people maximize ‘expected value’ by selecting actions that maximize potential reward while minimizing associated costs, including effort ‘costs’ and the opportunity cost of time. In MDD, differential weighing of costs and benefits are theorized mechanisms underlying changes in goal-directed cognition and may contribute to symptom heterogeneity.

**Methods:**

We used the Effort Foraging Task to quantify cognitive and physical effort costs, and patch leaving thresholds in low effort conditions (reflecting perceived opportunity cost of time) and investigated their shared versus distinct relationships to clinical features in participants with MDD (*N* = 52, 43 in-episode) and comparisons (*N* = 27).

**Results:**

Contrary to our predictions, none of the decision-making measures differed with MDD diagnosis. However, each of the measures was related to symptom severity, over and above effects of ability (i.e. performance). Greater anxiety symptoms were selectively associated with *lower* cognitive effort cost (i.e. greater willingness to exert effort). Anhedonia and behavioral apathy were associated with increased physical effort costs. Finally, greater overall depression was related to decreased patch leaving thresholds.

**Conclusions:**

Markers of effort-based decision-making may inform understanding of MDD heterogeneity. Increased willingness to exert cognitive effort may contribute to anxiety symptoms such as worry. Decreased leaving threshold associations with symptom severity are consistent with reward rate-based accounts of reduced vigor in MDD. Future research should address subtypes of depression with or without anxiety, which may relate differentially to cognitive effort decisions.

## Background

### Goal-directed behavior in major depressive disorder

Individuals with major depressive disorder (MDD) can experience challenges with goal-directed behavior, including reduced motivation due to symptoms such as apathy, anergia, and anhedonia, as well as reduced cognitive function. Decisions about which goals to pursue and which actions to take to achieve them, can be understood in terms of costs and benefits. People maximize ‘expected value’ by selecting actions that maximize potential reward while minimizing associated costs. Effort-based decision making involves minimizing cognitive and physical effort costs (Rigoux & Guigon, 2012; Salamone, Correa, Yang, Rotolo, & Presby, 2018; Shenhav et al., 2017; Shenhav, Botvinick, & Cohen, 2013; Walton, Rudebeck, Bannerman, & Rushworth, 2007), as well as the opportunity cost of time (often also emphasized in value-based decision-making, Constantino & Daw, 2015).

Under effort- and value-based decision-making disruption accounts of MDD symptoms, changes in goal-directed behavior in depression can come from multiple causes, for example, differences in representing either the benefits or the costs of potential actions. The present study focuses on cognitive and physical effort costs, as well as opportunity costs (i.e. reward rate), to understand how differences in these components of goal-directed behavior relate to clinical features of MDD.

Both cognitive and physical effort-based decision-making have been reported to differ in MDD, though findings have been mixed. MDD has been associated with decreased willingness to exert cognitive effort relative to comparison groups in some studies (Ang, Gelda, & Pizzagalli, 2023; Vinckier et al., 2022; Westbrook et al., 2022) though not in others (Barch et al., 2023; Tran, Hagen, Hollenstein, & Bowie, 2021). Willingness to exert physical effort has been found to be decreased in MDD relative to comparison groups in some studies (Berwian et al., 2020; Cléry-Melin et al., 2011; Treadway, Bossaller, Shelton, & Zald, 2012; Vinckier et al., 2022; Wang et al., 2022; Yang et al., 2014; Zou et al., 2020), though not in others (Cathomas et al., 2021; Sherdell, Waugh, & Gotlib, 2012; Tran et al., 2021; Wang et al., 2022; Yang et al., 2021).

### Dissociating between cognitive and physical effort costs

Both cognitive and physical effort-based decision-making appear to be associated with MDD features and may underlie certain MDD symptoms, though findings have been mixed. Importantly, MDD is highly heterogeneous in terms of variation in symptom domains and severity across individuals. This may contribute to inconsistent findings with respect to diagnostic group differences and associations with clinical features. MDD presentation encompasses many different symptoms, and decision-making mechanisms have many different components (including reward sensitivity, effort costs, task ability). Gaining traction on mechanistically informed treatments will require precise computational measures of decision-making to tease apart their specific relationships to precise symptom measures.

Initial studies measuring both effort types within-participants found differential relationships between cognitive and physical effort decisions and symptoms (Tran et al., 2021; Vinckier et al., 2022), suggesting potential applications to characterizing MDD heterogeneity. Studies measuring each effort type separately have reported decreased willingness to exert physical effort associated with symptom severity (i.e. anhedonia) (Sherdell et al., 2012; Tran et al., 2021; Yang et al., 2014), while others reported no relationship to symptom severity (e.g. not related to depression, anhedonia, apathy, (Ang et al., 2023; Barch et al., 2023; Hershenberg et al., 2016; Vinckier et al., 2022). For cognitive effort, some studies report associations with symptom severity (i.e. global functioning, Tran et al., 2021; Westbrook et al., 2022) while others do not (e.g. not related to depression, anhedonia, apathy, Ang et al., 2023; Barch et al., 2023; Hershenberg et al., 2016; Vinckier et al., 2022). It remains unclear which symptoms map onto which component decision processes, and how shared or distinct these mappings are between cognitive and physical effort.

### Hypothesized symptom relationships

Multiple symptom MDD domains have been proposed to relate to value- and effort-based decision-making. Subjective reward rate, reflecting the opportunity cost of time, is proposed to drive the vigor of actions, represented via midbrain dopamine tone (Niv, Daw, Joel, & Dayan, 2007). Drawing on this, Huys, Daw, and Dayan (2015) proposed that physical anergia and psychomotor slowing symptoms of depression may be caused by reduced subjective reward rate representations. Relatedly, reduced willingness to exert physical effort to obtain rewards has been proposed as a mechanism underlying anhedonia and apathy symptoms (Cooper, Arulpragasam, & Treadway, 2018; Husain & Roiser, 2018; Pessiglione, Vinckier, Bouret, Daunizeau, & Le Bouc, 2018). Grahek, Shenhav, Musslick, Krebs, & Koster (2019) proposed that changes in motivational processes, resulting in reduced willingness to exert cognitive effort, may underlie, in part, reduced cognitive function associated with MDD, challenging the standard assumption that reduced cognitive function reflects reduced cognitive control capacity (Millan et al., 2012; Rock, Roiser, Riedel, & Blackwell, 2014; Snyder, 2013). This is of particular importance because reduced cognitive function in MDD contributes to disability (Jaeger, Berns, Uzelac, & Davis-Conway, 2006) and often does not improve with otherwise effective anti-depressant treatments (Halahakoon & Roiser, 2016; Rosenblat, Kakar, & McIntyre, 2016). By the cognitive effort-based decision-making account, interventions to improve cognitive function would focus on boosting motivation and target willingness to engage control, rather than cognitive control ability (e.g. computerized cognitive training) as suggested by the reduced capacity account.

The variable prevalence of these symptoms across studies may contribute to mixed findings. For example, reduced motivation may be minimal or absent in some individuals with MDD (Ang, Lockwood, Apps, Muhammed, & Husain, 2017; Nakonezny, Carmody, Morris, Kurian, & Trivedi, 2010). In addition, certain symptom domains of depression may show a differential relationship to effort relative to others. Anxiety (the most common MDD comorbidity, Kessler et al., 1996) symptoms such as rumination and worry may require cognitive effort (e.g. sampling for replay and planning (Bedder, Pisupati, & Niv, 2023) and anxiety has been related to increased effortful model-based planning (Gillan, Kosinski, Whelan, Phelps, & Daw, 2016). Anxiety has also been linked to increased cognitive effort exertion to maintain performance in the face of increased attentional demands posed by threat-related stimuli (Eysenck, Derakshan, Santos, & Calvo, 2007). Additionally, social anxiety may be associated with enhanced motivation to exert cognitive effort in social contexts (such as a psychology experiment, Hunter, Bornstein, & Hartley, 2018). It therefore may be important to account for anxiety heterogeneity and relate cognitive effort-based decision making to specific depression symptom expression profiles (or subtypes), as some have suggested (Gagne, Zika, Dayan, & Bishop, 2020; Lynch, Gunning, & Liston, 2020).

### Experiment overview

The goal of the current study was to quantify multiple components of effort-based decision-making and decompose their contributions to MDD symptom expression profiles. Each component measured (cognitive and physical effort cost, cognitive task ability, subjective opportunity cost of time) has been proposed as an underlying mechanism of specific MDD symptoms. To test these accounts, we had participants who met diagnostic criteria for MDD (most in-episode) and demographically matched comparison participants with no psychiatric diagnoses complete the cognitive and physical Effort Foraging Task (Bustamante et al., 2023). To our knowledge, all previous MDD studies used explicit tasks in which participants choose between low-effort/low-reward and high-effort/high-reward options. We hypothesized that the Effort Foraging Task, which measures effort avoidance more implicitly by inferring the cost of effort from foraging behavior, would be less contaminated by demand characteristics that bring about changes in what participants value about effort (e.g. try to please the experimenter, Orne, 1962). Reduced control over demand characteristics in explicit tasks, in turn, may contribute to mixed findings. While we hypothesized the Effort Foraging Task would yield more valid findings regarding the relationship between MDD and willingness to exert effort, it could also reasonably be the case that this task may tap into distinct aspects of effort-based decision making than the tasks used in the extant literature (e.g. implicit versus explicit processing).

In the Effort Foraging Task, participants choose between harvesting a depleting patch, or traveling to a new patch, which is costly in time and effort. Participants completed the 3-Back level of an N-Back working memory task (Nystrom et al., 2000) in the high cognitive effort condition, the 1-Back level in low cognitive effort condition. Participants completed a larger number of rapid keypresses in the physical high effort condition and a smaller number of presses in the low physical effort condition. Analyses focused on ‘exit thresholds’, the reward value at which the participant decided to exit the current patch. The exit threshold reveals the point of equivalence in the tradeoff between the cost of harvesting with diminishing rewards and the cost of traveling to a new patch, and this is captured by a foraging-theory model (the Marginal Value Theorem, MVT, Charnov, 1976). According to the MVT, exit thresholds should reflect subjective average reward rate (i.e. opportunity cost of time), and this comports with human behavior (Constantino et al., 2017; Constantino & Daw, 2015; Lenow, Constantino, Daw, & Phelps, 2017). Based on this patch-leaving behavior, a foraging-theory-based computational model quantified individual differences in the ‘cost’ of effort. The longer a participant delayed leaving the patch in high versus low effort conditions (i.e. relatively lower the exit threshold) the larger their inferred effort cost parameter. Average exit thresholds in low effort blocks were used to assess overall foraging strategy, putatively reflecting subjective opportunity cost of time. Lastly, we assessed effortful travel task ability using accuracy and reaction times. We aimed to tease apart the influences of each of these effort decision-making components on clinical features of MDD by examining (i) diagnostic group differences, (ii) associations with overall depression severity, and (iii) associations with symptom severity.

Based on previous findings and theoretical work we predicted cognitive effort costs would be increased in the MDD group and related to cognitive function symptoms (i.e. subjective cognitive complaints relative to baseline, Grahek, Everaert, Krebs, & Koster, 2018; Grahek et al., 2019). Based on previous findings with explicit tasks, we predicted physical effort cost would be increased in the MDD group and related to anhedonia (Sherdell et al., 2012; Tran et al., 2021; Yang et al., 2014). Following Huys et al. (2015) we hypothesized that the MDD group would differ in foraging strategy, exhibiting lower exit overall thresholds, and that this would relate to physical anergia/slowing.

## Methods

### Study overview

#### Participants

97 participants volunteered for the study and gave informed consent as approved by the Rutgers University Institutional Review Board (67 MDD, mean = 26.9 years, s.d. = 11.1, 18–61; 30 comparison, mean = 27.1 years, s.d. = 9.64, 19–59, further details in online Supplementary S.I. section 1, Fig. S.I. S1). Groups were matched on key demographic variables (online Supplementary S.I. section 1.2). We oversampled MDD participants to maximize power to detect continuous symptom relationships to task behavior within this group. The comparison sample size was then selected to be adequate to detect group differences in behavior. Power analysis indicated that we could detect a medium effect size for group differences and symptom relationships with 80% power (online Supplementary S.I. section 2). All participants completed a detailed clinical assessment in session 1, but 7 MDD and 3 comparison participants opted not to return. Clinical symptom ratings and self-report analysis included data from the 60 MDD and 27 comparison participants who returned for the second (task) session. 50 MDD participants were currently depressed (43 with task data), 6 were in partial remission and 4 were in full remission (all with task data, details in online Supplementary S.I. section 3). 32 MDD participants used psychotropic medication while 28 did not.

#### Clinician ratings and self-reports

The Structured Clinical Interview for DSM-5 (First, 2015) confirmed assignment of MDD and/or co-morbid anxiety diagnosis in the MDD group (and absence of exclusionary diagnoses in both groups, online Supplementary S.I. section 1), as well as whether participants were currently depressed (or in partial or full remission). Depression symptoms were assessed using the Hamilton Depression Rating Scale (HAMD, Hamilton, 1960), Brief Psychiatric Rating Scale (Overall & Gorham, 1962), MGH Cognitive and Physical Functioning Questionnaire (Fava, Iosifescu, Pedrelli, & Baer, 2009), Patient Heath Questionnaire-9 (PHQ-9; Kroenke, Spitzer, & Williams, 2001), Generalized Anxiety Disorder-7 (Spitzer, Kroenke, Williams, & Lowe, 2006), Snaith–Hamilton Pleasure Scale (Nakonezny et al., 2010), Apathy Motivation Index (Ang et al., 2017), Adult Temperament Questionnaire Effortful Control subscale (Evans & Rothbart, 2007), and Need for Cognition scale (Cacioppo, Petty, & Kao, 1984, online Supplementary S.I. section 4, Table S.I. S1).

Symptom severity was measured using confirmatory factor analysis to combine clinician-rated and self-report measures of the following symptoms: anhedonia, anxiety, appetite symptoms, behavioral apathy, emotional apathy, social apathy, cognitive function symptoms, depressed mood/suicidality, and physical anergia/slowing, as well as for trait effortful control and need for cognition (see online Supplementary S.I. section 5, Tables S.I. S2, and S3). Items were assigned based on what each scale was validated to measure. Assigned items were *z* scored and averaged to compute a symptom severity score in the MDD group only. Items with an inter-item correlation below 0.2 were eliminated (multilevel package, item.total function, Bliese, Chen, Downes, Schepker, & Lang, 2022). Internal consistency was computed using Cronbach's alpha (online Supplementary Table S.I. S2, ltm package, cronbach.alpha function, Rizopoulos, 2006). Factors with alpha*<*0.6 were excluded from further analysis (i.e. emotional apathy, appetite symptoms). The resulting items were then applied to compute confirmatory factor scores for (1) MDD only, which was the focus of our analysis, and (2) all participants to test generalizability of effects.

### Effort foraging task

In the Effort Foraging Task participants harvested apples in virtual orchards (online Supplementary Fig. S.I. S2, as described in Experiment 2 of Bustamante et al. (2023)). On each foraging trial the participant visits a ‘patch’ which can be harvested by pressing the down arrow key once to yield rewards (apples, converted to a monetary bonus to be incentive compatible). The marginal return decreases with each successive harvest. The initial reward from a patch was drawn from a normal distribution *N*(15,1) and subsequent harvests were a product of the previous reward and a decay rate (drawn from a distribution beta distribution, *β*(14.90873, 2.033008), mean = 0.88). The smallest reward for harvesting was 0.5 apples and participants were not prevented from persisting in extracting 0.5 apples from the patch. At any point the participant can travel to a new patch by pressing the right arrow key, which has replenished rewards, but it takes time and effort to travel there (online Supplementary Fig. S.I. S3). The cognitive effort manipulation was the N-Back working memory task (1- and 3-Back levels, also used in Tran et al., 2021; Westbrook et al., 2022). The physical effort manipulation was rapid key pressing (also used in Berwian et al., 2020; Tran et al., 2021; Treadway et al., 2012; Treadway, Buckholtz, Schwartzman, Lambert, & Zald, 2009; Wang et al., 2022; Yang et al., 2014; Yang et al., 2021) with the non-dominant pinky finger (50 or 100% of an individually calibrated maximum). Patches were presented block-wise in counterbalanced order (online Supplementary S.I. section 6). Blocks varied only in their (explicitly instructed) effort requirement (timing held constant, environment specifications in Table 1). Reaching a new patch was not dependent on performance. Participants had to reach a performance criterion during training to begin foraging (training and instructions in online Supplementary S.I. section 7).

**Table 1.** Foraging environment parameters and results of best threshold simulation

A. Environment parameter	Value
Harvest time	2 s
Travel time	20 s
Cognitive Travel Tasks	1-Back, 3-Back (10 trials)
Physical Travel Tasks	Larger (100% max), Smaller (50%) number of presses
Block duration	7 min
Number blocks per condition	2
Number of conditions	4
Total Number of blocks	8
Initial reward Decay rate (*κ*)	*N*(15,1) *β*(14.13, 2.03), mean = 0.88
B. Best threshold simulation	Value
Best threshold (*ρ*, apples)	4.65
Reward rate (apples/second)	2.32
Number of harvests *μ*	9.95
Number of harvests s.d.	2.01

A: column 1: environment parameter, column 2: value. Participants completed eight 7-minute blocks. B: Best exit threshold policy identified in Bustamante et al. (2023), rows indicate best threshold from simulation, reward rate achieved with best threshold (apples per second), mean and standard deviation of the number of harvests it took to reach the best threshold.

We followed a subset of exclusions validated in Bustamante et al. (2023) that most impede estimates of effort costs (online Supplementary S.I. section 8). Participants were excluded if they missed the response deadline on many foraging trials (1 MDD participant excluded missed 49.5%) or had very few exit trials (1 MDD participant was excluded for 1 high effort physical and 3 high effort cognitive exits, 1 MDD participant was excluded from cognitive effort analyses for 2 cognitive high effort exits). The final sample included in behavioral analyses was 52 MDD participants (53 MDD participants for physical effort) and 27 comparison participants. We confirmed there were no diagnostic group differences among participants included in task-based analyses.

### Marginal value theorem (MVT) model

The MVT predicts a forager should leave a patch when the instantaneous reward rate falls below the long-run average (Charnov, 1976; Constantino & Daw, 2015). Travel costs were estimated using a hierarchical Bayesian logistic model. For each trial (*t*), the model compared the expected reward on the next harvest (*R*_*e*,*t*_, Eq. (1)) against the condition-specific exit threshold (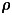
_condition_). Expected reward was based on the last reward multiplied by the mean depletion rate (*κ*). The first harvest of a patch, being forced, was excluded from analysis.1
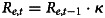


Using the difference of these to determine whether to harvest (1) or exit (0) via a softmax function (with inverse temperature, *β*, Eq. (2)).2
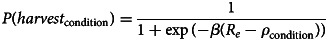


The cost of travel in high effort blocks (*c*_high effort_) was expressed as the marginal increase in cost of travel (*c*_low effort_ + *c*_high effort_) from low effort blocks, to control for any biases common to both conditions (e.g. variation in overall exit thresholds). Exit thresholds (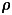) were taken as fixed per-condition, determined by the total rewards (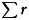), total amount of time (number of harvest periods, *T* = condition duration/harvest time) and total travel costs (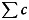, sum over total times travelled in a condition) across all blocks of a condition.

where,3
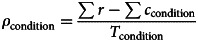


Individual and group-level parameters were estimated using Markov Chain Monte Carlo sampling (using cmdstanr, Stan, 2021). We used trace plots, 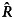 diagnostic statistics, and posterior predictive checks to assess model fit (online Supplementary S.I. section 9). We compared goodness of fit between diagnostic groups with an unpaired *t* test on participants' log posterior likelihoods (online Supplementary S.I. section 10). We compared participants' overall exit thresholds to the best threshold found by simulation (online Supplementary S.I. section 12).

For model-agnostic analyses, we used linear mixed-effects regression to predict log transformed expected reward (Eq. (4)) on exit trials by an intercept term and effort level term separately for each effort type.4
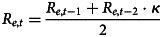


The first harvest of a patch was excluded from analysis, and on the second harvest of a patch we used the last reward multiplied by the depletion rate.

### Analysis overview

All subsequent analyses used point estimates (mean) from participant-level effort costs and applied them in frequentist tests to control for potential confounding variables and conduct multiple-comparison corrections. All analyses were conducted in the R language (many using the stats package, RCoreTeam, 2015). The HAMD total score was used to assess major depressive episode severity in the past week (herein ‘overall depression’). We verified that results matched using self-reported depression (PHQ-9) due to concerns with HAMD validity (Bagby, Ryder, Schuller, & Marshall, 2004; Gibbons, Clark, & Kupfer, 1993; Ma et al., 2021). Our focus was on MDD symptom severity (*z* scored for all analyses). To ensure remitted status was not driving key effects, we repeated all analyses zooming in on the current depressed group (excluding remitted participants). To test if effects were generalizable across the sample, we repeated analyses zooming out to all participants. We verified that no key results differed when controlling for psychotropic medication use (binary variable).

#### Diagnostic group differences

We tested for diagnostic group differences in cognitive or physical effort costs, controlling for high-effort task performance (3-Back D’ or % larger number of presses completed), years of education, age, and BMI (body mass index, for physical effort) using linear regression. To confirm hierarchical shrinkage did not bias results, we also fit group differences for all parameters directly within the MVT model (online Supplementary S.I. section 9). Additionally, we tested for a fatigue-like effect emerged within a block, and whether this differed by group (online Supplementary S.I. section 11).

#### Symptom associations with model parameters

Within the MDD group, we tested overall depression severity effects on cognitive or physical effort costs using linear regression, controlling for years of education, age, BMI (for physical effort) and high-effort task performance. Therefore, observed symptom associations are over and above effects of travel task ability. Next, we decomposed overall depression effects on effort costs into specific symptoms in a series of regression models. Because of mutual correlations between symptoms, we used multiple comparisons correction within a series of symptom models (FDR, 7 tests for each effort cost). We conducted a comparison of correlations to confirm specificity of observed relationships to effort type (cocor package) (Diedenhofen & Musch, 2015; Meng, Rosenthal, & Rubin, 1992). Because we allowed trial-wise variation to be captured by the inverse temperature parameter, we tested whether this parameter was correlated with symptom severity within the MDD group controlling for age and years of education.

#### Additional task measures

Participants may have differed in their ability to complete the required effort, which could confound decision-making differences and/or relate to symptoms. This is especially relevant for the cognitive task, which was not calibrated to individual ability. We addressed this by controlling for performance in symptom regression. We also examined whether performance was associated with (i) diagnostic group, (ii) overall depression, and (iii) symptom domains (online Supplementary S.I. section 13).

Some depression symptoms are theorized to arise from reduced subjective reward rate representations (Huys et al., 2015), which decrease vigor (Niv et al., 2007). We tested whether overall exit thresholds (from low effort conditions, which were least confounded by effort sensitivity) were associated with (i) diagnostic group, (ii) overall depression, or (iii) symptom domains (online Supplementary S.I. section 13.2).

## Results

### Demographic and clinical characteristics

Diagnostic groups were matched on gender, race, age, parental education, household income, and childhood income (also within participants included in behavioral analyses, online Supplementary Table S.I. S4). The comparison group had more years of education than the MDD group (online Supplementary Table S.I. S4), so it was included as a covariate in all analyses. Depression severity varied widely in the MDD group (online Supplementary Fig. S.I. S4). The MDD group scored higher on all symptom domains except emotional apathy. Need for cognition did not differ between groups, while effortful control was higher in comparisons (online Supplementary Fig. S.I. S5).

### Sensitivity to effort manipulations

On average, participants avoided effort (group-level posterior effort costs non-overlapping with zero, online Supplementary Table S.I. S5). The model converged (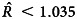
for all parameters, online Supplementary Fig. S.I. S6) and the observed probability of harvesting, across participants and trials, fell within the posterior predictive distribution (*pd >* 0.384). Simulated data recapitulated the empirical group-level change in exit threshold and overall exit threshold (online Supplementary Fig. S.I. S7) as well as the probability of exiting by expected reward level relative to individual overall exit thresholds (online Supplementary Fig. S.I. S8). We found no conclusive evidence for or against a correlation between cognitive and physical effort costs (mean = 0.053, 95% HDI = −0.240, 0.345, online Supplementary Table S.I. S5, discussed in the context of Experiment 2, Bustamante et al. (2023), in online Supplementary S.I. section S14).

### Effort costs relationships to clinical features

#### Diagnostic group differences

**Figure 1.**
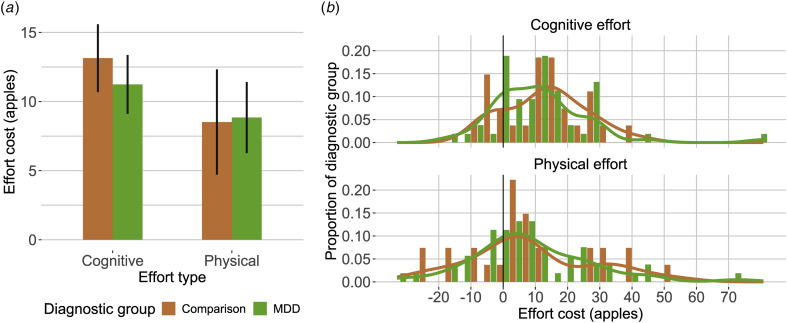
Effort cost by diagnostic group and effort type. (a) mean and standard error of the mean of individual differences in effort cost (*y* axis) by effort type (*x* axis). (b) individual differences histograms, *x* axis indicates effort cost (larger values indicate more effort avoidance), *y* axis indicates proportion of diagnostic group.

We predicted effort costs by diagnostic group, controlling for high-effort task performance, education, age, and BMI (for physical effort). There were no group differences in either effort cost (Fig. 1, cognitive: *p* *>* 0.70, physical: *p* *>* 0.77), even when controlling for psychotropic medication use (online Supplementary Table S.I. S6), and excluding remitted MDD participants (cognitive: *p* > 0.47, physical: *p* > 0.97). To ensure shrinkage in the hierarchical model did not obscure a group difference, we directly fitted diagnostic group differences for all MVT model parameters and found no differences in any of the parameters, even when excluding remitted MDD participants (online Supplementary Table S.I. S7). There were no group differences in the model-agnostic measure of effort sensitivity (online Supplementary S.I. section 16, Fig. S.I. S9), nor in fatigue-like effects (online Supplementary S.I. section 17). We computed the log posterior likelihoods per participant and found no significant difference between diagnostic groups, suggesting comparable goodness of fit (online Supplementary Fig. S.I. S7, *t* = 0.56, *df* = 36.28, *p* *>* 0.58). Consistent with prior samples we found a minority of participants who had negative effort costs, consistent with effort seeking, but no significant difference between the groups (online Supplementary S.I. section 15).

#### Overall depression severity

**Figure 2.**
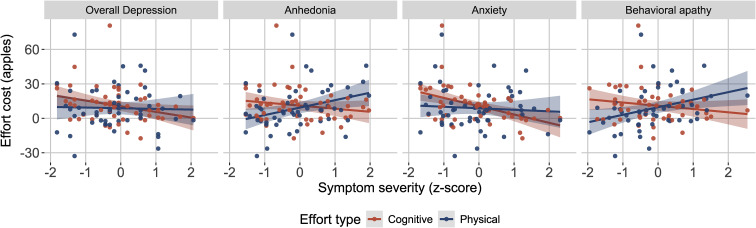
Effort costs relationships to individual MDD symptom domains. Blue indicates cognitive effort and red indicates physical effort. *y* axes: effort costs from MVT model, *x* axes: symptom severity (*z* scores) for overall depression (Hamilton Depression Rating Scale Total), anhedonia, anxiety, and behavioral apathy (MDD group only).

Surprisingly, overall depression severity was associated with *decreased* cognitive effort cost (*p* *<* 0.030, Fig. 2, Table 2). We found no reliable association with physical effort costs (Table 2) however, the correlation magnitudes between the two types of effort were not significantly different (*z* = −1.42, *p* = 0.156). Results were maintained when using self-reported depression (PHQ-9) as the overall severity measure (online Supplementary Table S.I. S8), and when controlling for medication use (online Supplementary Table S.I. S6). However, the cognitive effort cost relationship was not maintained when restricting the analyses to current MDD participants (online Supplementary Table S.I. S9). Additionally, we found no reliable association with inverse temperature (online Supplementary Table S.I. S10). Next, we identified which symptom domains contributed to the cognitive effort cost relationship to overall depression, and whether physical effort cost was related to any symptom domain.

**Table 2.** Symptom effort cost regressions (MDD group only)

Symptom	Estimate	s.e.	*t*	*p*	*p adjusted*	*R*^2^	adj. *R*^2^
*A. Cognitive effort cost, MDD*							
Overall depression	−0.37	0.16	−2.24	0.030*		0.120	0.045
Anhedonia	−0.17	0.15	−1.18	0.245	0.286		
Anxiety	−0.50	0.13	−3.70	0.001	0.004*	0.246	0.182
Behavioral apathy	−0.19	0.15	−1.28	0.205	0.286		
Social apathy	0.01	0.15	0.06	0.950	0.950		
Cognitive function symptoms	−0.30	0.14	−2.08	0.043	0.150		
Depressed mood/suicidality	−0.26	0.17	−1.56	0.126	0.253		
Physical anergia/slowing	−0.22	0.15	−1.48	0.145	0.253		
*B. Physical effort cost, MDD*							
Overall depression	0.04	0.17	0.21	0.833			
Anhedonia	0.42	0.14	2.93	0.005	0.035*	0.193	0.103
Anxiety	−0.04	0.15	−0.26	0.793	0.793		
Behavioral apathy	0.39	0.15	2.71	0.010	0.035*	0.173	0.082
Social apathy	0.14	0.15	0.93	0.355	0.492		
Cognitive function symptoms	0.27	0.16	1.67	0.103	0.240		
Depressed mood/suicidality	0.13	0.17	0.81	0.422	0.492		
Physical anergia/slowing	0.16	0.16	1.00	0.324	0.492		
*C. Cognitive effort cost, MDD, controlling for anxiety*							
Anhedonia	−0.06	0.14	−0.40				
Behavioral apathy	−0.06	0.14	−0.40	0.689	0.991		
Social apathy	0.01	0.13	0.07	0.947	0.991		
Cognitive function symptoms	0.001	0.17	0.01	0.991	0.991		
Depressed mood/suicidality	0.13	0.19	0.67	0.508	0.991		
Physical anergia/slowing	0.06	0.16	0.39	0.696	0.991		

(A) Predicting cognitive effort cost from overall depression severity, and each symptom domain, controlling for cognitive task performance (3-Back D’) years of education and age. (B) Predicting physical effort cost from overall depression severity, and each symptom domain, controlling for physical task performance (% larger number of presses completed), BMI, years of education and age. (C) Predicting cognitive effort cost from each symptom domain, controlling for anxiety, cognitive task performance (3-Back D’) and age (* indicates *p* *<* 0.05, FDR correction within symptom models). *R*^2^ and adjusted *R*^2^ displayed for significant models. All variables were scaled as input to the regressions.

#### Symptom specific relationships

We fitted regression models to estimate symptom domain relationships to effort costs while controlling for high-effort travel task performance, age, education, and BMI for physical effort (see online Supplementary Fig. S.I. S10 for heatmap of symptoms and task correlations). Anxiety was related to *decreased* cognitive effort cost (Fig. 2, Table 2), and this pattern was maintained in the current MDD group, across all participants (online Supplementary Table S.I. S9), and when controlling for medication use (online Supplementary Table S.I. S6). Given our inclusion of participants with comorbid anxiety disorders and prior literature relating anxiety to increased effortful model-based strategy, we tested whether statistically accounting for anxiety would reveal any other any other symptoms relationships to cognitive effort cost when and found no reliable relationships (Table 2).

We examined symptom associations with physical effort costs. Within the MDD group, anhedonia and behavioral apathy were associated with *increased* physical effort costs (Table 2). These effects were maintained (1) when controlling for medication (though not after FDR correction, online Supplementary Table S.I. S6), (2) in the current MDD group only (online Supplementary Table S.I. S9). In all participants, there was no reliable association with behavioral apathy, and the association with anhedonia was significant only before FDR correction. There was a significant difference in the correlation magnitudes of cognitive and physical effort cost with anxiety (*z* = −2.15, *<*0.031), anhedonia (*z* = −2.71, *p* *<* 0.007) and behavioral apathy (*z* = −2.69, *p* *<* 0.007) within the MDD group.

### Additional task measures

#### Travel task performance

All the effort cost symptom analyses are over and above any effects related to travel task ability (i.e. task performance), which was controlled for. We examined diagnostic group differences and performance associations to symptoms directly (online Supplementary S.I. section 18). There were minimal diagnostic group differences in performance; however, the MDD group completed a lower percentage of keypresses in the high physical effort condition and responded faster on average on the cognitive task (online Supplementary Fig. S.I. 11, Table S.I. S11). Neither cognitive nor physical performance was reliably related to overall depression. While anxiety symptoms were associated with cognitive effort costs, they were not associated with cognitive task performance (online Supplementary Fig. S.I. S12, S.I. section 18). Anhedonia symptoms were related to a lower percentage of completed keypresses in the low (but not high) physical effort condition (*p* *<* 0.010). Cognitive task performance did not predict cognitive effort cost, nor did physical performance predict physical effort cost, suggesting effort decisions and execution are dissociable in this task (online Supplementary Fig. S.I. S12).

#### Overall exit threshold

**Figure 3.**
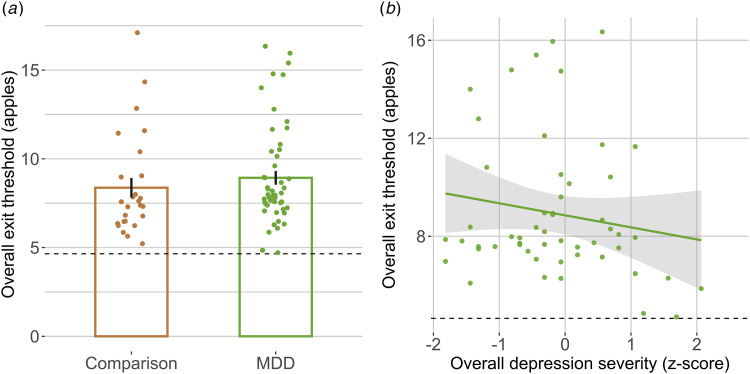
Relationship of individual MDD symptom domains with overall exit threshold (MDD group only). (a) No diagnostic group differences (*x* axis) in overall threshold (*y* axis, apples, estimated from low effort conditions). Bar indicates group means, error bars indicate standard error of the mean, points indicate mean overall exit threshold per participant (i.e. random effects coefficients from linear regression model). (b) Lower overall exit threshold (*y* axes) was significantly related to overall depression severity (*x* axes, Hamilton Depression Rating Scale Total *z* score). Dashed line indicates best threshold policy, linear regression line for MDD group only.

We did not observe diagnostic group differences in overall exit thresholds in a model controlling for age and education (*p* *>* 0.339). However, when medication was a covariate, medication use was associated with lower exit thresholds, and MDD group membership was associated with higher thresholds (online Supplementary Table S.I. S6). Overall exit thresholds were lower in participants with greater overall depression (Table 3, but not for other symptom domains), and this association was significant within the current depressed group (Fig. 3, online Supplementary Table S.I. S12). The overall depression effect was maintained when controlling for medication (and additional effects were found for depressed mood/suicidality and physical anergia/slowing, online Supplementary Table S.I. S6). Overall, this pattern is consistent with theories of reduced subjective reward rate representation associated with depression (Huys et al., 2015).

**Table 3.** Symptom overall exit threshold regressions (MDD group only)

Symptom	Estimate	s.e.	*t*	*p*	*p adjusted*
*Overall exit threshold, MDD*					
Overall depression	−0.33	0.16	−2.09	0.042*	
Anhedonia	−0.26	0.14	−1.80	0.078	0.109
Anxiety	−0.34	0.14	−2.43	0.019	0.066
Behavioral apathy	−0.30	0.15	−2.09	0.042	0.074
Social apathy	−0.10	0.14	−0.73	0.470	0.470
Cognitive function symptoms	−0.21	0.15	−1.44	0.157	0.184
Depressed mood/suicidality	−0.40	0.15	−2.68	0.010	0.066
Physical anergia/slowing	−0.32	0.14	−2.20	0.033	0.074

Predicting individual differences in overall exit thresholds (log, from low effort conditions) by symptom severity, controlling for age and years of education (* indicates *p* *<* 0.05, FDR correction within symptom models). Overall depression model *R*^2^ = 0.085 and adjusted *R*^2^ = 0.029. All variables were scaled as input to the regressions.

## Conclusions

This cross-sectional study compared cognitive and physical effort-based decision-making using computational-model-derived parameters from the Effort Foraging Task (Bustamante et al., 2023), within-participant, in a heterogeneous group of participants with MDD and non-psychiatric comparisons. We found novel and important dissociable relations between symptom dimensions of MDD and cognitive versus physical effort. Our results corroborate several computational theories of depression and support breaking depression down into symptom domains and to examining components of effort- and value-based decision-making within-participants.

### Diagnostic group differences

We predicted MDD would be associated with increased effort avoidance for both cognitive (Ang et al., 2023; Hershenberg et al., 2016; Vinckier et al., 2022; Westbrook et al., 2022) and physical (Berwian et al., 2020; Cléry-Melin et al., 2011; Treadway et al., 2012; Vinckier et al., 2022; Yang et al., 2014; Zou et al., 2020) effort. Contrary to our hypotheses, we did not observe significant group differences in effort costs. This aligns with null results in other studies of cognitive (Barch et al., 2023; Tran et al., 2021) and physical effort avoidance (Cathomas et al., 2021; Sherdell et al., 2012; Tran et al., 2021; Wang et al., 2022; Yang et al., 2021). There were minimal task *performance* differences, except the MDD group had faster N-Back reaction times and completed fewer keypresses in the high physical effort condition. Travel task performance and effort costs were not correlated in this sample, suggesting they are dissociable in this task.

### Symptom associations with effort costs

Greater overall depression severity in the MDD group was associated with greater willingness to exert cognitive effort, with no such relationship for physical effort. Although the comparison of correlations did not indicate specificity with respect to effort type, anxiety symptom severity accounted for the cognitive effort cost association, whereas physical effort cost was not reliably related to anxiety. The comparison of correlations indicated effort type specificity. To our knowledge no effort-based decision-making studies have reported decreased cognitive effort avoidance associated with MDD, suggesting unaccounted anxiety variation might contribute to inconsistent findings.

The negative association between anxiety and cognitive effort cost is consistent with reports of increased model-based planning associated with anxiety in unselected samples (Gillan et al., 2016; Hunter et al., 2018). Increased cognitive effort exertion in anxiety might act as a compensatory mechanism to maintain performance amid threat-related attentional demands (Eysenck et al., 2007). Clinically, increased willingness to exert cognitive effort may contribute to anxiety symptoms such as rumination and worry through increased planning and replay (Bedder et al., 2023). Higher effort tasks might reduce anxious thoughts due to increased cognitive load (presumably via distraction). This is consistent with research showing reduced momentary anxiety during a high relative to low cognitive effort task (Vytal, Cornwell, Arkin, & Grillon, 2012).

Despite aiming to minimize demand characteristics, the social context of the experiment may have motivated anxious participants to exert effort (rather than expressing underlying preferences). An online study with the Effort Foraging Task found anxiety was associated with increased cognitive effort cost, opposite to present findings (Bustamante et al., 2023). However, the study was conducted in a large, unselected sample with self-reported symptoms, complicating translation to this clinical sample (another online study also did not report cognitive effort avoidance relationships to anxiety and depression, Patzelt, Kool, Millner, & Gershman, 2019).

Anhedonia and behavioral apathy were significantly associated with increased physical effort costs within the MDD group, when controlling for psychotropic medication, and the current MDD group, but not across all participants. The anhedonia association is consistent with some reports, (Sherdell et al., 2012; Tran et al., 2021; Yang et al., 2014), but not others, (Berwian, Walter, Seifritz, & Huys, 2017; Cathomas et al., 2021; Cléry-Melin et al., 2011; Vinckier et al., 2022; Wang et al., 2022; Yang et al., 2021; Zou et al., 2020). Behavioral apathy has previously not been found to be reliably related to physical effort avoidance (Cathomas et al., 2021; Cléry-Melin et al., 2011; Vinckier et al., 2022). Willingness to exert cognitive effort was not related to anhedonia or behavioral apathy and comparison of correlations demonstrated differential relationships by effort type.

These findings support measuring both cognitive and physical effort decision-making function markers, which may inform heterogeneity or subtypes of MDD. Two lines of evidence suggest observed relationships between behavior and symptoms were driven by motivation rather than ability. First, all analyses relating effort costs to symptom severity accounted for ability (i.e. high effort performance) such that reported effects are over and above effects related to ability. Second, we found no reliable direct relationships between ability and MDD symptoms. The differential associations between anxiety versus anhedonia/behavioral apathy may suggest dissociable symptom dimensions caused by cognitive versus physical factors respectively (however, the present study was limited in teasing apart cognitive versus somatic forms of anxiety). Future studies can test whether anhedonia stems more from physical factors such as peripheral symptoms (e.g. fatigue), whereas anxiety is driven more by cognitive factors.

The Effort Foraging Task was developed to measure effort preferences more implicitly to increase validity. Therefore, methodological differences from previous (explicit) effort tasks may have contributed to the identification of symptom relationships, which has been mixed in other studies. On the other hand, implicit and explicit decisions may reflect unique effort-based decision-making aspects that differentially relate to MDD. It remains a question whether results from this task are more valid or if they tap into a different dimension of effort-based decision-making than the explicit task literature. If this were the case, it would enhance the novel contribution but also limit generalizability of the findings. Nevertheless, this work opens a new avenue for understanding how effort-based decision making relates to depression and other psychiatric symptoms, and how variations in task and modeling approaches affect such relationships.

### Subjective reward rate in depression

The MVT predicts that decisions to leave a patch reveal perceived environmental quality (i.e. opportunity cost of time), and this is supported by evidence in humans (e.g. lower thresholds under acute and chronic stress, in persons with Parkinson's, in persons with opioid use disorder, associations with dopamine receptor availability, Constantino et al., 2017; Constantino & Daw, 2015; Ianni et al., 2023; Lenow et al., 2017; Raio et al., 2021). Following Huys et al. (2015), we hypothesized MDD would be associated with lower exit thresholds, reflecting reduced average reward expectations. Few studies have linked psychiatric symptoms to sensitivity to opportunity cost of time, though one found an association with self-reported apathy (Nair et al., 2023). Overall exit thresholds did not differ by group (though a group effect emerged when accounting for psychotropic medication use in an unexpected direction) but were decreased in MDD participants with greater overall depression severity (also when excluding remitted participants). This suggests a difference in value-based decision-making associated with the severity of MDD symptoms, such that environments may be subjectively represented as less rewarding, reducing goal-directed behavior and vigor (Huys et al., 2015; Niv et al., 2007).

### Limitations

These results leave open the question of whether observed symptom associations generalize to other psychiatric conditions or other effort-based decision measures (e.g. tasks, ecological momentary assessment). To determine if the association between anxiety and cognitive effort cost is specific to MDD, future studies could include participants with primary clinical anxiety disorders. Another limitation is that sample size of the remitted depressed group did not allow for comparison with other groups. Heterogeneity in psychotropic medication use is another limitation, given neurotransmitter effects on aspects of cognition measured in the task. However, key effects were robust to excluding remitted participants and controlling for psychotropic medication use. The cross-sectional design limits understanding causality between symptoms and task behavior. Longitudinal designs could distinguish state versus trait influences on cognitive control and effort-based decision making and their interaction with symptoms.

### Clinical implications

Ultimately insights from this research may inform interventions to increase willingness to exert effort for individuals experiencing challenges with goal-directed behavior due to psychiatric disability. Therapies that use cognitive restructuring to target physical effort perception might be effective for addressing anhedonia symptoms. Therapies for depression may target subjective reward rate, possibly through pharmacological dopamine manipulations (Niv et al., 2007). For applications to anxiety, the causal direction of the association with cognitive effort cost should be established. Does reduced cognitive effort cost cause anxiety symptoms, or does anxiety cause the pattern observed (i.e. benefits of distraction)? The tendency to exert cognitive effort could be leveraged as a strength in treating anxious depression (e.g. positive fantasizing, more cognitively effortful therapies, novel therapeutic applications using distraction, Besten, van Tol, van Rij, & van Vugt, 2023).

## Supporting information

Bustamante et al. supplementary material Bustamante et al. supplementary material

## Data Availability

The analysis code and partial data are openly available at the Open Science Framework (OSF) at https://doi.org/10.17605/OSF.IO/TZJ28. Note that participants did not consent to the public sharing of select variables connected to others in the dataset.

## Supplementary material

The supplementary material for this article can be found at https://doi.org/10.1017/S0033291724002691
