# Supplementary material for: Major depression symptom severity associations with willingness to exert effort and patch foraging strategy
Source: Psychol Med. 2024 Dec 2;54(15):4396–407. doi: 10.1017/S0033291724002691 (PMC11650159; doi:10.1017/S0033291724002691)
Supplement: Bustamante et al. supplementary material [file S0033291724002691sup001.pdf]

Supplementary Information

Contents

- S.I. section 1 Study overview
- Figure S.I. 1 Inclusion and exclusion diagram
- S.I. section 1.2 Diagnostic group matching
- S.I. section 2 Power analysis
- S.I. section 3 Major depressive episode criteria
- S.I. section 4 Clinician ratings and self reports
- Table S.I. 1 Clinical interview and self-report battery
- S.I. section 5 Symptom confirmatory factor analysis
- Table S.I. 2 Confirmatory symptom factors alpha and diagnostic group differences
- Table S.I. 3 Items for confirmatory symptom factors
- Figure S.I. 2 Effort Foraging Task Diagram
- S.I. section 6 Effort Foraging Task counterbalancing
- S.I. section 7 Task instructions and training
- Figure S.I. 3 Travel task methods
- S.I. section 8 Foraging behavior analysis exclusions
- S.I. section 9 MVT model additional methods
- S.I. section 10 MVT model evaluation
- S.I. section 11 Fatigue effects group differences methods
- S.I. section 12 Simulation to find best threshold

- S.I. section 13 Additional task measures methods
- S.I. section 13.2 Overall exit threshold
- Table S.I. 4 Demographic factors by diagnostic group
- Figure S.I. 4 MDD sample characteristics
- S.I. section 14 Relationship between cognitive and physical effort costs
- Figure S.I. 5 Symptom severity by diagnostic group
- Table S.I. 5 MVT group-level posterior distributions
- Figure S.I. 6 Model diagnostics
- Figure S.I. 7 Posterior predictive check
- Figure S.I. 8 Posterior predictive check probability of exit by expected reward level
- S.I. section 15 Diagnostic group differences in effort-seeking
- S.I. section 16 Model-agnostic sensitivity to effort manipulation
- S.I. section 17 Fatigue effects group differences results
- Table S.I. 6 Symptom regressions, control for psychotropic medication use, MDD
- Table S.I. 7 Diagnostic group difference MVT model
- Figure S.I. 9 Model-agnostic change in exit threshold by diagnostic group
- Table S.I. 8 Self-reported overall depression regression results
- S.I. section 18 Additional task measures results
- Table S.I. 9 Symptoms effort cost regressions, current MDD and all participants
- Figure S.I. 10 Task behavior symptom heatmap
- Table S.I. 10 Symptoms inverse temperature regressions, MDD group

- Figure S.I. 11 Travel task performance by diagnostic group
- Table S.I. 11 Diagnostic group differences in travel task performance
- Figure S.I. 12 Performance relationship to effort costs and symptoms
- Table S.I. 12 Overall exit threshold relationship to symptoms, current MDD and all participants

1 Study overview

The study was conducted at the Rutgers-Princeton Center for Computational Neuro-Psychiatry by trained clinical researchers. The study was approved by the Rutgers University Institutional Review Board. Participants were recruited in outpatient clinics at Rutgers University, as well as via Google Ads. We recruited participants with MDD confirmed using the Structured Clinical Interview for DSM-5 (SCID-5, First, 2015) and no co-occurring psychiatric conditions except for anxiety disorders, which were permitted. MDD participants in remission ("no significant symptoms during the past 2 months") and in partial remission ("symptoms are present but full criteria are not met, for a period less than 2 months") were permitted. We also recruited a demographically matched comparison group without any psychiatric diagnosis. The study was administered remotely via secure web-based software (Zoom, except for 5 MDD participants who came an in-person session) and participants were compensated $20 per hour and an Effort Foraging Task bonus of up to $10. On the first session a clinical interviewer administered the SCID-5 and participants completed self-report surveys. On the second session participants completed the Effort Foraging Task. In a third session participants completed tasks assessing reward sensitivity and cognitive control recruitment in response to efficacy manipulations, which will be reported separately.

1.1 Inclusion and exclusion criteria

The study inclusion criteria were 1) between the ages of 18-65, 2) has the capacity to provide informed consent, and 3) is fluent in English, 4) score of 6 or higher on the Wechsler Test of

Adult Reading (equivalent to standardized score of 53 - 60 for ages 18-64, Wechsler, 2001), 5) (MDD only) Meets DSM-5 criteria for MDD as confirmed by the SCID-5, (MDD only) if the participant is treated with anti-depressant medication, they are on stable treatment with this medication (i.e. no change in medication type, or substantial change in dose, for at least 4 weeks prior to participating in the study). The study exclusion criteria were 1) history of traumatic brain injury or head injury, 2) Intellectual disability or pervasive developmental disorder, 3) neurological disease, 4) has met DSM-5 criteria for a substance-use disorder within the last 6 months per the SCID-5 (with the exception of nicotine dependence, which was permitted), 5) received electroconvulsive therapy within the last 8 weeks, 6) left-handedness (due to keyboard set up for the Effort Foraging Task), 7) (comparisons only) meets DSM-5 criteria for any psychiatric diagnosis as confirmed by the SCID-5, 8) (comparisons only) current use of any psychotropic medication.

1.2 Diagnostic group matching

We used Pearson's Chi-squared test to compare categorical diagnostic group differences (gender, race, ethnicity, total household income, occupational status, relationship status, alcohol frequency, alcohol amount, caffeine amount, tobacco use), and Welch two sample unpaired t-test to compare continuous diagnostic group differences (age, childhood income, years of education for self and parents), using the chisq.test and t.test functions of the stats package in the R language, RCoreTeam, 2015). If diagnostic groups were not matched on a demographic variable, we included it as a covariate in our group difference and symptom relationships to task behavior analyses.

2 Power analysis

We conducted a power analysis with the current sample size (using the pwr package, Champely et al., 2020). For the symptom regressions, power analysis indicated we could detect a medium effect size with 80% power with the current sample of 52 MDD participants (F^2=0.254, Cohen's F^2 'medium' effect size between 0.15 and 0.35, J. Cohen, 1992). For the diagnostic

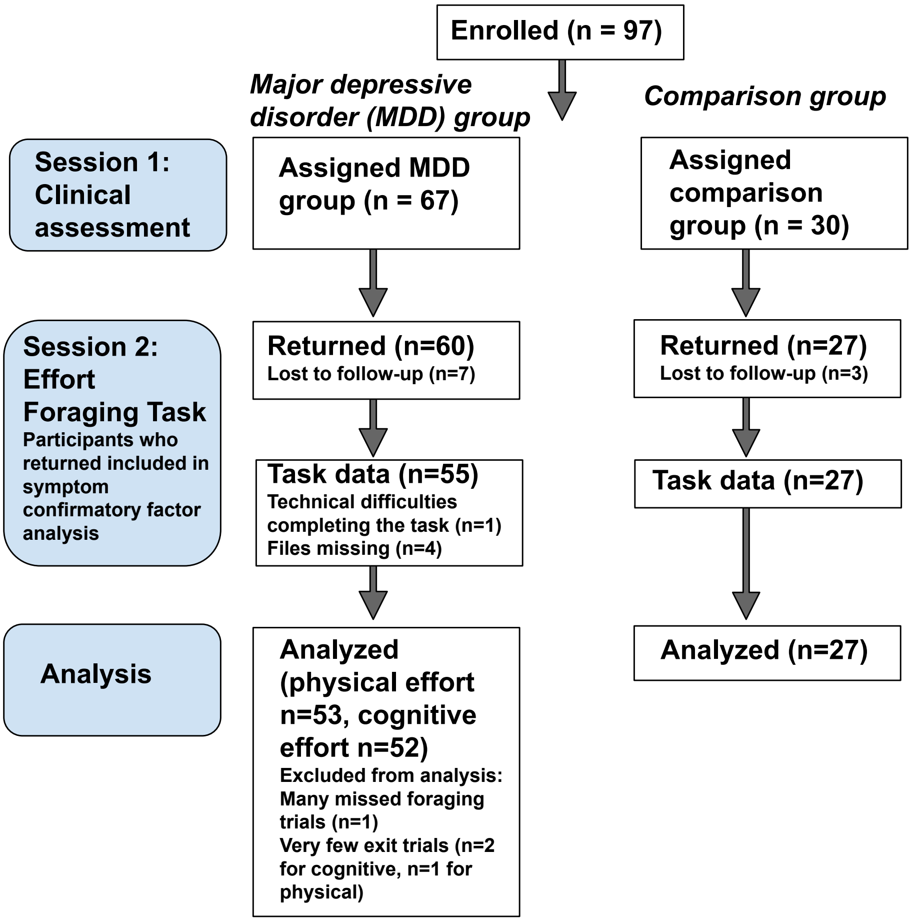

Figure S.I. 1: Inclusion and exclusion diagram. First column indicates experimental phase, session 1 clinical assessment, follow-up session 2 task measure, and number of participants analyzed. Second column indicates MDD group, third column indicates comparison group.

group differences, power analysis indicated we could detect a medium effect size with 80% power with the current sample of 52 MDD and 27 comparison participants (D=0.673, Cohen's D 'medium' effect size between 0.5 and 0.8, J. Cohen, 1992).

3 Major depressive episode criteria

Participants were assigned to the MDD diagnostic group based on the SCID-5, which considers the lifetime history for the MDD diagnosis, and symptoms in the past two months to establish whether a participant is 1) currently depressed, meeting criteria for a major depressive episode any time in the past month, 2) in partial remission, either experiencing some symptoms but not meeting full criteria for a major depressive episode, or there is a period lasting less than two months without significant symptoms, or 3) in full remission, during the past two months no significant symptoms. All participants in the MDD group have a lifetime history of depression, but varied in how many symptoms they experienced in the past week, as well as in the past two months. This dynamic variation in MDD symptom expression, if unaccounted for, may contribute to mixed results surrounding diagnostic group differences in effort-based decision making. This motivated us to focus on individual differences in symptom severity at the time of the study, rather than solely diagnostic group differences. The present study utilized the HAMD, which measures symptoms in the past week, along with the BPRS which measures symptoms in the past 2-3 days, as well as self-reports which asked about either the past few days, two weeks, or month. Therefore, currently depressed participants may have low scores on the HAMD (or BPRS) if they report fewer symptoms in the past week relative to the past month (during which they did meet full criteria for a major depressive episode). Low scores on the HAMD (or BPRS) would be expected for fully or partially remitted MDD participants, although scores could be higher for these participants if they are experiencing some symptoms but did not meet full criteria for a major depressive episode.

4 Clinician ratings and self reports

The Structured Clinical Interview for DSM-5 (SCID-5, First, 2015) was used to confirm assignment of MDD, co-morbid anxiety, and comparison groups, and that participants met study diagnostic inclusion and exclusion criteria. The clinical interviewer used responses in the SCID-5 to rate the severity of different symptoms in the past week via the the Hamilton Depression Rating Scale (HAMD, Hamilton, 1960) and to determine whether MDD participants were currently depressed, or in partial or full remission. This was followed by the semi-structured interview the Brief Psychiatric Rating Scale (Overall and Gorham, 1962) to assess current psychiatric-symptom severity. Next participants completed self-report surveys to measure; cognitive function symptoms and physical anergia with the Massachusetts General Hospital Cognitive and Physical Functioning Questionnaire (Fava, Iosifescu, Pedrelli, and Baer, 2009), depression symptoms with the Patient Heath Questionnaire-9 (Kroenke, Spitzer, and Williams, 2001), anxiety symptoms with the Generalized Anxiety Disorder-7 (Spitzer, Kroenke, Williams, and Löwe, 2006), anhedonia symptoms with the Snaith–Hamilton Pleasure Scale (Nakonezny, Carmody, Morris, Kurian, and Trivedi, 2010), behavioral, emotional, and social apathy with the Apathy Motivation Index (Ang, Lockwood, Apps, Muhammed, and Husain, 2017). We also measured trait executive function with the Adult Temperament Questionnaire Effortful Control subscale (Evans and Rothbart, 2007), and trait cognitive control seeking with the Need for Cognition scale (Cacioppo, Petty, and Kao, 1984).

**Scale name**	**N items**	**Abbreviation**	**Time scale**
Hamilton Depression Rating Scale	21	HAM-D	Past week
Brief Psychiatric Rating Scale	24	BPRS	Last 2 weeks
MGH Cognitive and Physical Functioning Questionnaire	7	MGH-CPFQ	Last month
Patient Health Questionnaire-9	9	PHQ-9	Last 2 weeks
Generalized Anxiety Disorder-7	7	GAD7	Last 2 weeks
Apathy Motivation Index	14	AMI	Last 2 weeks
Snaith-Hamilton Pleasure Scale	13	SHAPS	Last few days
Adult Temperament Questionnaire-Effortful Control	19	ATQ-EC	Trait
Need for Cognition	18	NFC	Trait
**Total Self-report**	87		

Table S.I. 1: Clinical interview and self-report battery. Clinical interview and self-reports were completed in session 1 in the order listed in this table. Scales asking about similar timescales were grouped together.

5 Symptom confirmatory factor analysis

We performed confirmatory factor analysis and assigned items from clinician ratings and self-report into the following domains (using measures listed in Table S.I. 1, exact items in Table S.I. 3): anhedonia, appetite symptoms, anxiety, behavioral apathy, cognitive function symptoms, depressed mood, effortful control (trait), emotional apathy, need for cognition (trait), physical anergia/slowing, social apathy. Assigned items were z-scored and averaged to compute a symptom score in (1) the MDD group only and (2) all participants. Confirmatory factor scores for comparison participants did not include ratings from clinician measures (i.e., HAMD, and BPRS) and instead were the average z-score of all self-report items in a factor.

We tested whether diagnostic groups significantly differed in symptom intensity and cognitive control trait measures using t-tests (Table S.I. 2). For left skewed distributions (comparisons clustered on very low symptom scores for anxiety, cognitive function symptoms, depressed mood, and physical anergia/slowing) we confirmed the diagnostic group differences results were maintained using the non-parametric Wilcoxon rank sum test with continuity correction (wilcox.test function of the stats package in the R language, RCoreTeam, 2015).

**Symptom domain**	**alpha(95% CI)**	**N**	**MDD**	**Comp.**	***t***	**df**	***p***
Anhedonia	0.89(0.84-0.92)	15	0.132	-0.294	3.07	55.46	0.003
Anxiety	0.90(0.86-0.93)	10	0.197	-0.626	7.386	83.523	<0.001
Appetite symptoms	0.52(0.20-0.69)	3	0.091	-0.609	5.501	76.74	<0.001
Behavioral apathy	0.72(0.53-0.82)	8	0.186	-0.554	6.124	58.79	<0.001
Emotional apathy	0.54(0.30-0.68)	5	-0.049	0.137	-1.22	44.08	0.230
Social apathy	0.71(0.55-0.81)	5	0.149	-0.331	3.303	58.024	0.002
Cognitive function symp.	0.84(0.75-0.89)	5	0.257	-0.572	6.880	80.293	<0.001
Depressed mood	0.86(0.81-0.90)	8	0.106	-0.626	7.508	66.346	<0.001
Effortful control (trait)	0.84(0.76-0.88)	17	-0.177	0.393	-6.29	80.5	<0.001
Need for cognition (trait)	0.90(0.85-0.93)	17	-0.033	0.074	-0.794	58.151	0.430
Physical anergia/slowing	0.76(0.64-0.84)	7	0.157	-0.611	7.616	84.89	<0.001

Table S.I. 2: Confirmatory symptom factors alpha and diagnostic group differences. Column 1: symptom domain, column 2: Cronbach's alpha (95% confidence interval), column 3: number of items, column 4-8: MDD mean, Comparison mean, and t-statistic, degrees of freedom, p-value. Emotional apathy and appetite symptoms had low Cronbach's alpha scores and were not included in further analysis.

Category	Items	Scale
Anhedonia	Little interest or pleasure in doing things	PHQ-9
Anhedonia	I would enjoy my favorite television or radio program	SHAPS
Anhedonia	I would enjoy being with family or close friends	SHAPS
Anhedonia	I would find pleasure in my hobbies and pastimes	SHAPS
Anhedonia	I would be able to enjoy my favorite meal	SHAPS
Anhedonia	I would enjoy a warm bath or refreshing shower	SHAPS
Anhedonia	I would find pleasure in the scent of flowers or the smell of a fresh sea breeze or freshly baked bread	SHAPS
Anhedonia	I would enjoy seeing other people's smiling faces	SHAPS
Anhedonia	I would enjoy looking smart when I have made an effort with my appearance	SHAPS
Anhedonia	I would enjoy reading a book, magazine or newspaper	SHAPS
Anhedonia	I would enjoy a cup of tea or coffee or my favorite drink	SHAPS
Anhedonia	I would find pleasure in small things; e.g., bright sunny day, a telephone call from a friend	SHAPS
Anhedonia	I would be able to enjoy a beautiful landscape or view	SHAPS
Anhedonia	I would get pleasure from helping others	SHAPS
Anhedonia	I would feel pleasure when I receive praise from other people	SHAPS
Anxiety	Anxiety psychic	HAM-D
Anxiety	Anxiety - somatic	HAM-D
Anxiety	Anxiety	BPRS
Anxiety	Feeling nervous, anxious, or on edge	GAD-7
Anxiety	Not being able to stop or control worrying	GAD-7

Category	Items	Scale
Anxiety	Worrying too much about different things	GAD-7
Anxiety	Trouble relaxing	GAD-7
Anxiety	Being so restless that it's hard to sit still	GAD-7
Anxiety	Becoming easily annoyed or irritable	GAD-7
Anxiety	Feeling afraid as if something awful might happen	GAD-7
Behavioral apathy	How has your motivation/interest/enthusiasm been over the past month?	MGH-CPFQ
Behavioral apathy	Work and interests	HAM-D
Behavioral apathy	Self-neglect	BPRS
Behavioral apathy	I make decisions firmly and without hesitation.	AMI
Behavioral apathy	When I decide to do something, I am able to make an effort easily.	AMI
Behavioral apathy	I get things done when they need to be done, without requiring reminders from others.	AMI
Behavioral apathy	When I decide to do something, I am motivated to see it through to the end.	AMI
Behavioral apathy	When I have something I need to do, I do it straightaway so it is out of the way.	AMI
Emotional apathy	Emotional withdrawal	BPRS
Emotional apathy	I feel sad or upset when I hear bad news.	AMI
Emotional apathy	Based on the last two weeks, I would say I care deeply about how my loved ones think of me.	AMI
Emotional apathy	I feel bad when I hear an acquaintance has an accident or illness.	AMI
Emotional apathy	If I realize I have been unpleasant to someone, I will feel terribly guilty afterwards.	AMI
Social apathy	I start conversations with random people.	AMI
Social apathy	I enjoy doing things with people I have just met.	AMI

Category	Items	Scale
Social apathy	I suggest activities for me and my friends to do.	AMI
Social apathy	I go out with friends on a weekly basis.	AMI
Social apathy	I start conversations without being prompted.	AMI
Appetite	Somatic symptoms - gastro-intestinal	HAM-D
Appetite	Weight loss	HAM-D
Appetite	Poor appetite or overeating	PHQ-9
Effortful control (trait)	I am often late for appointments.	ATQ-EC
Effortful control (trait)	I often make plans that I do not follow through with.	ATQ-EC
Effortful control (trait)	I can keep performing a task even when I would rather not do it.	ATQ-EC
Effortful control (trait)	I can make myself work on a difficult task even when I don't feel like trying.	ATQ-EC
Effortful control (trait)	If I think of something that needs to be done, I usually get right to work on it.	ATQ-EC
Effortful control (trait)	I usually finish doing things before they are actually due (for example, paying bills, finishing homework, etc.).	ATQ-EC
Effortful control (trait)	When I am afraid of how a situation might turn out, I usually avoid dealing with it.	ATQ-EC
Effortful control (trait)	It's often hard for me to alternate between two different tasks.	ATQ-EC
Effortful control (trait)	When I am trying to focus my attention, I am easily distracted.	ATQ-EC
Effortful control (trait)	When interrupted or distracted, I usually can easily shift my attention back to whatever I was doing before.	ATQ-EC
Effortful control (trait)	It is very hard for me to focus my attention when I am distressed.	ATQ-EC

Category	Items	Scale
Effortful control (trait)	When I am happy and excited about an upcoming event, I have a hard time focusing my attention on tasks that require concentration.	ATQ-EC
Effortful control (trait)	Even when I feel energized, I can usually sit still without much trouble if it's necessary.	ATQ-EC
Effortful control (trait)	I can easily resist talking out of turn, even when I'm excited and want to express an idea.	ATQ-EC
Effortful control (trait)	I usually have trouble resisting my cravings for food drink, etc.	ATQ-EC
Effortful control (trait)	When I'm excited about something, it's usually hard for me to resist jumping right into it before I've considered the possible consequences.	ATQ-EC
Effortful control (trait)	When I see an attractive item in a store, it's usually very hard for me to resist buying it.	ATQ-EC
Need for cognition (trait)	I would prefer complex to simple problems.	NFC
Need for cognition (trait)	I like to have the responsibility of handling a situation that requires a lot of thinking.	NFC
Need for cognition (trait)	Thinking is not my idea of fun.	NFC
Need for cognition (trait)	I would rather do something that requires little thought than something that is sure to challenge my thinking abilities.	NFC
Need for cognition (trait)	I try to anticipate and avoid situations where there is a likely chance I will have to think in depth about something	NFC
Need for cognition (trait)	I find satisfaction in deliberating hard and for long hours.	NFC

Category	Items	Scale
Need for cognition (trait)	I only think as hard as I have to.	NFC
Need for cognition (trait)	I prefer to think about small daily projects to long term ones.	NFC
Need for cognition (trait)	I like tasks that require little thought once I've learned them.	NFC
Need for cognition (trait)	The idea of relying on thought to make my way to the top appeals to me.	NFC
Need for cognition (trait)	I really enjoy a task that involves coming up with new solutions to problems.	NFC
Need for cognition (trait)	Learning new ways to think doesn't excite me very much.	NFC
Need for cognition (trait)	I prefer my life to be filled with puzzles I must solve.	NFC
Need for cognition (trait)	The notion of thinking abstractly is appealing to me.	NFC
Need for cognition (trait)	I would prefer a task that is intellectual, difficult, and important to one that is somewhat important but does not require much thought.	NFC
Need for cognition (trait)	I feel relief rather than satisfaction after completing a task that requires a lot of mental effort.	NFC
Need for cognition (trait)	It's enough for me that something gets the job done; I don't care how or why it works.	NFC
Cognitive function symptoms	Distractibility (speech and actions interrupted by stimuli unrelated to the interview)	BPRS
Cognitive function symptoms	How has your ability to focus/sustain attention been over the past month?	MGH-CPFQ

Category	Items	Scale
Cognitive function symptoms	How has your ability to remember/recall information been over the past month?	MGH-CPFQ
Cognitive function symptoms	How has your ability to find words been over the past month?	MGH-CPFQ
Cognitive function symptoms	How has your sharpness/mental acuity been over the past month?	MGH-CPFQ
Cognitive function symptoms	Trouble concentrating on things, such as reading the newspaper or watching television	PHQ-9
Depressed mood	Depressed mood	HAM-D
Depressed mood	Feelings of guilt	HAM-D
Depressed mood	Suicide	HAM-D
Depressed mood	Depression	BPRS
Depressed mood	Suicidality	BPRS
Depressed mood	Guilt	BPRS
Depressed mood	Feeling down, depressed, or hopeless	PHQ-9
Depressed mood	Feeling bad about yourself - or that you are a failure or have let yourself or your family down	PHQ-9
Depressed mood	Thoughts that you would be better off dead, or of hurting yourself	PHQ-9
Physical anergia/slowing	Somatic symptoms - general	HAM-D
Physical anergia/slowing	Psychomotor retardation	HAM-D
Physical anergia/slowing	Motor retardation (slowed or reduced movements or speech)	BPRS
Physical anergia/slowing	How has your wakefulness/alertness been over the past month?	MGH-CPFQ

Category	Items	Scale
Physical anergia/slowing	How has your energy been over the past month?	MGH-CPFQ
Physical anergia/slowing	Feeling tired or having little energy	PHQ-9
Physical anergia/slowing	Moving or speaking so slowly that other people could have noticed. Or the opposite - being so figety or restless that you have been moving around a lot more than usual.	PHQ-9

Table S.I. 3: Items for confirmatory symptom factors. Column 1: symptom domain, column 2: items, column 3: measurement scale (abbreviations in Table S.I. 1).

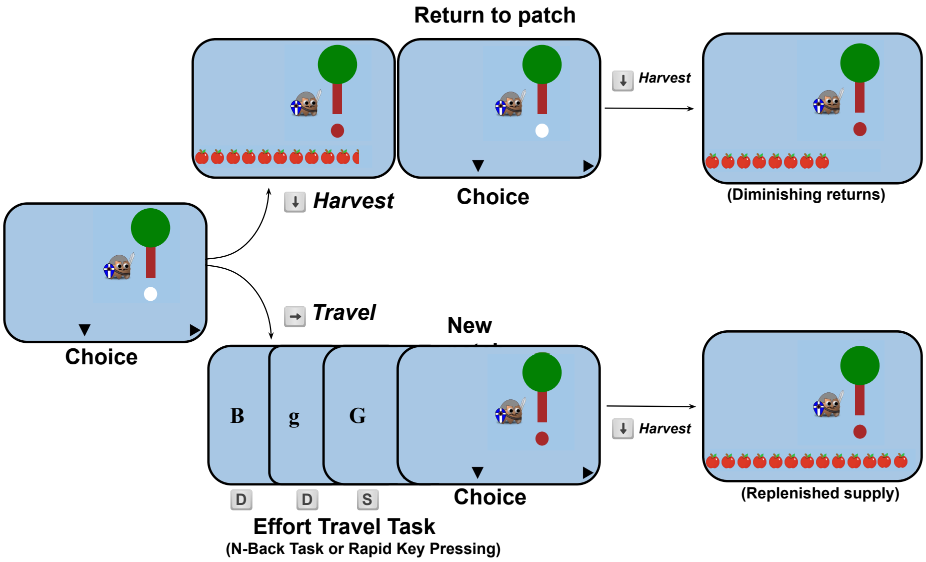

Figure S.I. 2: Effort Foraging Task Diagram. On each trial participants chose to harvest a virtual patch (apple tree) using the down arrow key, or travel to a new patch. Harvesting a patch yielded diminishing returns, whereas traveling to a new patch cost time and effort. Travel tasks were either the 1-Back or 3-Back levels of the N-Back task, or a smaller or larger number of rapid keypresses. Adapted from Figure 1 of Bustamante et al., 2023.

6 Effort Foraging Task counterbalancing

The order of cognitive and physical effort variants was counterbalanced across participants. Within blocks of an effort type, each effort level was tested once during the first half and once during the second half. Given that constraint, the effort level was fully counterbalanced, resulting in eight possible orders. Participants were assigned a block order using latin squares within each diagnostic group.

7 Task instructions and training

“Welcome to the experiment! Thank you for participating. This experiment will require you to press buttons on your keyboard repeatedly, applying varying amounts of physical effort. If you have any history of any sort of hand injury or pain with typing (e.g., which could make either fast button pressing or stretching your hand uncomfortable) please do not complete this task. You must wait a minimum of 5 seconds before you are able to progress to a new slide of

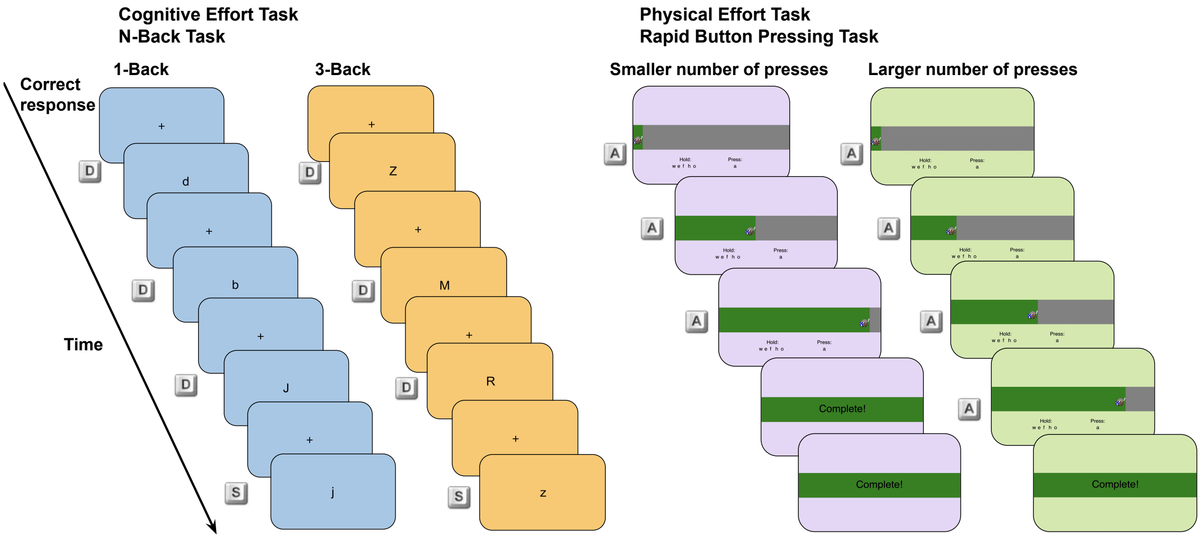

Figure S.I. 3: Travel task methods. Left panel: cognitive effort, N-Back working memory task. Participants responded whether the letter on the screen was the same (‘s’ key) or different (‘d’ key). The background color differed for the high effort (3-Back, orange) and low effort (1-Back, blue) conditions. Key icons next to each screen indicate the correct response. Right panel: physical effort, rapid key-pressing task. Participants rapidly pressed the ‘a’ key while holding down the ‘w’, ‘e’, ‘f’ (left hand), ‘h’ and ‘o’ keys (right hand). Pressing the ‘a’ key moved the avatar rightwards and filled up the grey horizontal bar with green. When participants reached the goal number of presses ‘Complete!’ appeared in the horizontal bar and participants waited for the remainder of the travel time. The background color differed for the high effort (smaller presses, 50% of maximum, purple) and low effort (larger presses, 100% of maximum, green) conditions. Adapted from Figure 2 of Bustamante et al., 2023.

instructions. You will know you can click to a new slide when the "Next" button changes."

"Welcome to the Apples Game! For your completion of this task, you will receive a potential bonus between $0 and $5. Please read the instructions carefully. There will be a quiz at the end of these instructions to check your understanding. In this game, you will make choices that earn you money. Imagine you are a farmer, and you are harvesting apples from trees in your multiple orchards. On every trial within an orchard, you will see a tree: To HARVEST the tree, press the down arrow key with your right hand. Do this when the circle below the tree is white. When you harvest the tree it gives you apples. These apples are worth real money that you will earn on top of the money for participating in this study. Now, try harvesting the tree three times in a row. Press the HARVEST key to collect apples from the tree. [Press "Next" to practice using the HARVEST key 3x]"

"The more times you harvest a tree, the fewer apples it gives you! On any trial, instead of accepting the number of apples the tree is giving you, you have the option to TRAVEL to a new tree. To TRAVEL to a new tree press the right arrow key with your right hand. Do this when the circle below the tree is white. Now, harvest the tree once and travel from one tree to another. Do this three times. Press the TRAVEL key to move to a new tree. [Press "Next" to practice using the TRAVEL key 3x]"

"Different trees give you different number of apples at the start. Exactly how many apples a tree starts with changes from tree to tree. The starting number of apples for a tree is NOT RELATED to how the tree looks or which orchard you are in. HARVESTING takes some time but earns you apples. TRAVELING takes longer, and you cannot harvest apples during traveling. But it brings you to a new tree with a full supply. You have to decide how to spend your limited time in an orchard – harvesting or traveling. You have to HARVEST each new tree once before traveling away from it. If you take too long to make a choice you will miss a turn and see this message. The more turns you miss, the less time you have to harvest apples, and you will earn less apples. That is most of what you have to know to be a great farmer. Let's go through a short practice orchard in the Apples Game."

Then participants completed a 1.5-minute orchard with no travel task. Then the learned one of the effortful travel tasks (order of effort types counterbalanced).

7.1 Foraging task training

In the task began with training the travel task for the first effort cost variant for a particular participant (this could be the cognitive or physical effort task). Next came instructions for the foraging task in general (without mentioning the effortful travel requirement), and participants completed a practice block (90 seconds) of the foraging task with no travel task. Then participants were instructed that they would have to complete the effortful travel task when traveling, and they completed two practice blocks (one per effort level, 90 seconds each). Then, participants completed the main foraging task for the first travel task type (4 blocks, 7 minutes per block, with self-paced breaks between blocks). After completing all the blocks of the first travel task, participants began training on the second travel task. They were instructed that they would continue to play the foraging task, but the travel task had changed. They practiced the foraging task with the second travel task type (one practice block per effort level, 90 seconds each). Finally, they completed the main foraging task for the second travel task type (4 blocks, 7 minutes per block).

7.2 Rapid key-pressing task training

In Experiment 1 key-press training began with a calibration phase (three rounds) to determine the maximum number of presses participants were able to complete in the travel time (7.5 seconds of effort task time). A counter was displayed on the center of the screen showing how many presses a participant had made. The instructions suggested participants were being compared to others, and encouraged them to press as fast as possible, each round they were encouraged to press faster than they had the previous rounds. Then we used each participant's mean number of presses across rounds as their 'maximum number'. We enforced a minimum 'maximum number' value of 20 presses. The Larger Number of Presses condition tasked participants with completing 100% of their maximum, and the Smaller Number of Presses condition tasked participants with completing 50% of their maximum. Participants were told that there was a larger and smaller number, but not what that number was or how it was determined. Then participants practiced a single effort level. Effort level order was counterbalanced. Practice for an effort level began with a single mini-block the duration of the

foraging travel time. Then participants had to complete 5 mini-blocks reaching the required number of presses to move on. This was meant to establish the expectation that participants would perform well on the travel task, even though there were no incentives or punishments associated with travel task performance during the foraging task.

7.2.1 N-Back working memory task

The N-Back task was performed as part of foraging task during travel between trees. In the N-Back task letters are displayed on screen in a sequence. Participants judged whether the stimulus that is currently on the screen matches the stimulus they saw a number of screens back (N-Back). On every trial, participants responded whether the letter was a match ("s" key) or non-match ("d" key) to the letter on the previous screen (1-Back case) or three screens before (3-Back case). A trial began with a fixation cross (for 250 milliseconds) followed by the letter on screen (for 500 milliseconds) followed by a blank screen (for 950 milliseconds, total trial duration = 1.7 seconds). During the travel period, 10 letters were presented, of which, 2 or 3 were targets (letter matches letter N-Back) and 2 or 3 were lures (matches current letter but not in position N-Back). The number of targets and lures were selected randomly each time an N-Back stimulus sequence was generated. We only used consonants to prevent participants from using mnemonics (letters were: 'B', 'C', 'D', 'F', 'G', 'H', 'J', 'K', 'M', 'N', 'P', 'Q', 'R', 'S', 'T', 'V', 'W', 'X', 'Y', 'Z'), and half of the letters were presented in upper case and the other half lower case to prevent participants using iconic memory (J. D. Cohen et al., 1994).

7.2.2 N-Back working memory task training

We trained the N-Back task extensively to try to bring participants to highest possible levels of performance and minimize automaticity differences (in which some participants would have more experience with the N-Back or similar tasks, making the task less effortful for them compared to someone with little experience). Participants had to reach a performance criterion to move on from training. After being instructed on the task participants began practice for one of the effort levels (counterbalanced). First, they completed two extended blocks

(50 trials with a self-paced break up to 45 seconds between) with feedback about error type (types of feedback: “non-match”, “missed match”, “no response”, displayed in red font for 800 ms after the trial). Then they performed one extended block without any feedback (50 trials).

We tasked participants with completing a set number of mini-blocks with high accuracy to begin the foraging task. We did so to establish the expectation that participants had to exert effort when they chose to travel while foraging. A mini-block was classified as successful when the participant saw no error feedback (large black dot), after which they were told they were moving on to the next mini-block. The error feedback was displayed when participants made two consecutive errors (including omission errors). If they did see one or more error feedback symbols, they had to repeat that mini-block. They had to successfully complete 8 mini-blocks of the 1-Back task, and 12 mini-blocks of the 3-Back task. This training also ensured that participants could adequately perform the task. Participants had self-paced breaks in between mini-blocks (up to 60 seconds).

8 Foraging behavior analysis exclusions

Of the 60 MDD and 27 comparison participants, 1 MDD participant did not complete the Effort Foraging Task due to technical difficulties with their keyboard. All other participants completed the task, however technical difficulties with the experiment server caused 4 missing data files from the MDD group. We followed a subset of exclusions validated in Bustamante et al. (2023) that most interfere with estimating effort costs. First, participants were excluded if they had very few exit trials within an effort type, making their data under-powered for estimating exit thresholds, and overly deterministic for logistic regression, which are the basis of the effort cost measures (2*SD below the mean, $<$8.82 trials). As a result 1 MDD participant was excluded from analysis for the whole task (1 exit in high effort physical and 3 exits in high effort cognitive condition) and 1 MDD participant was excluded from the cognitive effort analyses (2 exits for the cognitive high effort condition). Second, participants were excluded from the task if they missed the response deadline on many foraging trials (2*SD

above the mean, >15.05%, 1 MDD participant excluded who missed 49.5% of trials) which may reflect low engagement with the task or challenges meeting the response deadline. Ultimately, this affects the interpretability of MVT estimates (e.g., experienced harvest time longer than for other participants, fewer apples per second). The final sample included in behavioral analyses was 52 MDD participants (53 MDD participants in the physical effort condition) and 27 comparison participants.

9 MVT model additional methods

Because we are investigating individual differences in effort costs at the condition level, used a factorial model in which the MVT threshold is taken as fixed per-condition, determined by the overall rewards and delays in each condition and a per-condition effort-cost parameter. Thus, the model omits trial-by-trial learning of the threshold, and instead formally absorbs any such variation into the softmax choice stochasticity. We believe this simplification is warranted because the condition-wise effort costs of interest aggregate over per-trial threshold variability, and because we encouraged asymptotic behavior through pre-training and using a stable foraging environment throughout.

There were five parameters in the model, the inverse temperature (β, which controls the noise of the softmax choice function, with lower values indicating more noisy effects of rewards and thresholds on choices), the cognitive low ($c_{\text{cog low effort}}$) and high effort costs ($c_{\text{cog high effort}}$), and the physical low ($c_{\text{phys low effort}}$ and high effort costs ($c_{\text{phys high effort}}$). The model included a full covariance matrix of the parameters (5-by-5 matrix) which consists of a correlation matrix and a scale (standard deviation) matrix. Parameters were drawn from a multi-variate Gaussian distribution. We used the covariance matrix to estimate the correlation between individual differences in high cognitive and physical effort costs. Model priors were centered at zero and variances were selected to accommodate the magnitude of group-level posterior distributions from the original Effort Foraging Task study (Experiment 2, Bustamante et al., 2023). The prior distributions for group-level effects were $c_{\text{low effort}} \sim \mathcal{N}(0, 25)$, $c_{\text{high effort}} \sim \mathcal{N}(0, 15)$, $\beta \sim \mathcal{N}(0, 1)$. The prior on random effects variances were $c_{\text{low effort}} \sim \mathcal{N}(0, 25)$,

$c_{\text{high effort}} \sim \mathcal{N}(0, 15)$, $\beta \sim \mathcal{N}(0, 1)$. The prior on the correlation matrix was unbiased as to the presence or absence of a correlation (LKJ Correlation Distribution prior=1, Lewandowski, Kurowicka, and Joe, 2009). Individual participant parameters and their group-level distributions were estimated using Markov Chain Monte Carlo sampling, implemented in Stan with the CmdStanR package (4,000 samples, 2,000 warm-up samples, across four chains, Stan Development Team, Stan, 2021). Convergence was assessed by visually inspecting model traces, and ensuring the $\hat{R}$ convergence diagnostic statistic was below 1.1. We also simulated the MVT model to estimate the best exit threshold with respect to reward and time given the foraging environment parameters. To test for diagnostic group (g) differences, we fitted a Hierarchical Bayesian MVT model in which each of the 5 group-level parameters (p) had a diagnostic group effect ($\beta_{g,p}$) added to it. For a participant (i) in diagnostic group (g_i) each participant-level parameter p_i was the sum of the group-level parameter p and the diagnostic group effect ($p_i = p + \beta_{g,p} * g_i$, where $g_{MDD}, g_{Comparison} \in 0.5, -0.5$). The diagnostic group effect parameters ($\beta_{g,p}$) for low and high effort travel costs had a prior distribution $\mathcal{N}(0, 5)$, and inverse temperature had a prior distribution $\mathcal{N}(0, 0.5)$, values greater than zero indicate higher effort costs in the MDD relative to comparison group. To confirm the remitted and partial remitted participants did not change the results, we also used this model to test for group differences of the comparison group to the currently depressed subset of the MDD group.

10 MVT model evaluation

We used several methods to evaluate the MVT model fit. We inspected trace plots to ensure mixing between chains, and the $\hat{R}$ convergence diagnostic was below 1.1 for all parameters (using the rhat and mcmc_trace functions from the bayesplot package in R, Gabry et al., 2024). We conducted a posterior predictive check to confirm the fitted model captured foraging decisions. For each of 8,000 MCMC samples, for all trials across the entire dataset, the model sampled from the posterior predictive distribution from a Bernoulli distribution, generating a set of harvest (1) or exit (0) choices (using the bernoulli_logit_rng function in Stan, Team, 2021). We examined the correspondence of the posterior predictive samples to

the empirical data. We tested whether the empirical probability of stay choices (across all participants and all trials in the dataset) fell within the posterior predictive distribution (i.e., the simulated probability of stay choices across all trials for each MCMC sample). To do so, we computed the distance from the median simulated probability of staying for every MCMC sample, as well as the distance from the empirical data. We tested the probability that the distance of the empirical data to the simulated median was larger than the distances of the simulated data. Additionally, we visually compared the overall exit threshold, as well as the change in exit threshold by diagnostic group, in simulated versus empirical data. Similarly, we visually compared the probability of exiting the patch across expected reward levels, as well as the change in the probability of exiting the patch by effort level (high - low effort conditions) across expected reward levels.

We assessed the log posterior likelihood per participant and used an unpaired t-test to compare goodness of fit for the model between the diagnostic groups. To do so, we computed the sum of log likelihoods within each participant, for each MCMC sample (using the bernoulli_logit_lpmf function in Stan, Team, 2021). Then we aggregated across MCMC samples by exponentiating these values, summing them, and log-transforming them, resulting in one log posterior likelihood value per participant (reflecting the logarithm of the posterior probability of the model parameters given the observed data for a participant). A lack of diagnostic group difference in this metric would suggest comparable goodness of fit.

11 Fatigue effects group differences methods

To measure fatigue we used linear mixed-effects regression predicting the model-agnostic measure of expected reward (log(apples)) by the effort level interacted with exit number within a round (starting at one to the total number of exits for a round) separately for cognitive and physical effort. Random effects terms were the effort level and exit number within a round, but without the interaction term due to convergence issues. We reasoned that fatigue should increase each time the travel task was completed. Because there were self-paced breaks between rounds we did not look at fatigue across rounds. Following this simpler model, we

added the diagnostic group as a 3-way interaction with fatigue and effort level.

12 Simulation to find best threshold

We considered the best threshold found by simulation in Bustamante et al. (2023) for Experiment 2. To repeat the methods we used in that study, we simulated the best foraging threshold by creating a foraging environment with an agent with a fixed exit threshold and observing the resulting reward rate. We used a policy iteration algorithm to find the maximal reward rate for a given foraging environment. The foraging environment was defined by the following parameters from our experiments; the harvest time (2 seconds), travel time (8.33 seconds), the distribution of initial rewards to a tree $N(15, 1)$ distribution of the decay function (beta distribution, $\beta(14.90873, 2.033008)$). We assumed the agent knew the mean depletion rate (0.88 multiplied by the previous reward) and used this value to predict the expected reward on the current trial. If the predicted reward was less than or equal to the agent's threshold it exited the patch $R_e \leq \rho$, otherwise it harvested the patch which yielded reward. We simulated 840 'seconds' of foraging time for all experiments (though the result should be robust to duration). The simulation outputs were the 'best threshold' (threshold that yielded the highest reward rate, results vary slightly by simulation run), the resulting 'best reward rate', as well as the mean and standard deviation number of harvests to reach that exit threshold.

The agents' threshold parameter was initialized at 4 apples. For an iteration i, the threshold was set as the mean reward rate observed in iteration i-1, this allowed the threshold to gradually improve in terms of reward rate between iterations. The simulation stopped and the best threshold was determined based on the stopping threshold of a 0.001 apple per second improvement in reward rate on iteration i compared iteration i-1 (with a maximum of 200 iterations).

13 Additional task measures methods

13.1 Task ability

Using a series of regression models, we tested whether diagnostic groups differed on effortful travel task performance. For the cognitive (N-Back) task we tested for differences in accuracy, reaction time, and missed trials. Using linear regression, we predicted N-Back accuracy (D') by N-Back level interacted with diagnostic group. We used logistic mixed-effects regression to predict N-Back reaction times (log transformed) across all trials by a 4-way interaction between N-Back level, correct or incorrect response, target or non-target trial, and diagnostic group, controlling for age. We used logistic mixed-effects regression to predict the percent of missed N-Back trials by diagnostic group.

For the physical effort (rapid key-pressing) task we compared the groups on the required number of keypresses (determined during calibration) and the percent of keypresses completed during travel. Using linear regression, we predicted required keypresses by diagnostic group controlling for age. In a linear mixed effects regression, we predicted the percent of completed keypresses per travel interval by the effort level (smaller or larger number of presses) interacted with diagnostic group, controlling for age and BMI.

We tested whether cognitive and physical effort costs were dissociable from task ability (i.e., performance). Using data from all participants, in the first model we predicted cognitive effort cost by 1-Back and 3-Back D', controlling for age. In the second model we predicted cognitive effort cost by the change in D' from 1-Back to 3-Back (which in line with effort cost as a change score from low to high effort). In the third model we predicted physical effort cost by the percent of key presses completed in the larger number of presses, and the smaller number of presses condition, controlling for age and BMI.

13.2 Overall exit threshold

Overall exit threshold individual differences were estimated from a linear mixed effects regression model that predicted exit thresholds (log apples) in low effort orchards (which were least confounded by effects of effort) with effort type as a fixed effect, and random intercepts

per participant. To test for diagnostic group differences, we added diagnostic group to the regression. To test for relationships with depression symptoms in the MDD group only, we ran a series of linear regressions predicting overall exit threshold by i) overall depression, and ii) each of the symptom domains separately (7 tests), controlling for age. We corrected for multiple comparisons across symptoms (FDR, 7 tests). We repeated these analyses zooming in to the currently depressed MDD group only and zooming out to all participants.

	MDD		*Comparison*	
	**Mean**	**SD(range)**	**Mean**	**SD(range)**
Age (t=-0.083, df=57.2, p=0.934)				
	26.92	11.1(18-61)	27.11	9.64(19-59)
Age, behavioral analyses (t=-0.47, df=53.94, p=0.643)				
Childhood income (t=-2.35, df=61.73, p=0.022)				
	5.07	1.98(1-8)	6.00	1.52(3-9)
Childhood income, behavioral analyses (t=-2.12, df=62.94, p=0.038)				
Years education				
Mother (t=0.086, df=61.8, p=0.931)				
	15.13	3.41(5-20)	15.07	2.73(11-20)
Mother, behavioral analyses (t=0.46, df=62.41, p=0.645)				
Father (t=-1.17, df=61.93, p=0.245)				
	14.8	4.08(0-20)	15.8	3.26(12-20)
Father, behavioral analyses (t=-0.75, df=59.07, p=0.456)				
Self (t=-2.51, df=47.7, p<0.016*)				
	14.7	2.15(9-21)	16.0	2.28(13-20)
Self, behavioral analyses (t=-2.40, df=49.17, p<0.020)				
Education-self correlation with effort costs				
	**Correlation**	***t***	**df**	***p***
Cognitive effort cost, MDD group				
	-0.03	-0.24	50	0.809
Cognitive effort cost, all participants				
	0.09	0.80	77	0.427
Physical effort cost, MDD group				
	0.15	1.07	51	0.290
Physical effort cost, all participants)				
	0.06	0.53	78	0.599
	MDD		*Comparison*	

	MDD		*Comparison*	
	**N**	**%**	**N**	**%**
	**N**	**%**	**N**	**%**
Gender (χ^2=2.39, *df*=2, *p*=0.303)				
Gender, behavioral analyses (χ^2=3.75, *df*=2, *p*=0.154)				
Female	38	63.3%	15	55.6%
Male	19	31.7%	12	44.4%
Non-binary	3	5%	0	0%
Race (χ^2=1.66, *df*=4, *p*=0.800)				
Race, behavioral analyses (χ^2=1.63, *df*=4, *p*=0.803)				
American Indian/Alaska Native	0	0%	0	0%
Asian	13	21.7%	8	29.6%
Black or African American	8	13.3%	3	11.1%
Native Hawaiian/Pacific Islander	0	0%	0	0%
White	27	45%	13	48.1%
Other or prefer not to say	6	10%	2	7.41%
More than one race	6	10%	1	3.7%
Ethnicity (χ^2=1.41, *df*=2, *p*=0.495)				
Ethnicity, behavioral analyses (χ^2=1.71, *df*=2, *p*=0.424)				
Latino or Hispanic	8	13.3%	4	14.8%
Not Latino or Hispanic	49	81.7%	23	85.2%
Other or prefer not to say	3	5%	0	0%
Total household income (χ^2=9.44, *df*=7, *p*=0.222)				
Total household income, behavioral analyses (χ^2=9.02, *df*=7, *p*=0.251)				
Less than $25,000	12	20%	1	3.7%
$25,000 to $34,999	7	11.7%	3	11.1%
$35,000 to $49,999	8	13.3%	2	7.41%
$50,000 to $74,999	9	15%	8	29.6%
$75,000 to $99,999	5	8.33%	5	18.5%

	MDD		***Comparison***	
	**N**	**%**	**N**	**%**
$100,000 to $149,999	6	10%	3	11.1%
$150,000 to $199,999	0	0%	1	3.7%
$200,000 or more	4	6.67%	2	7.41%
Prefer not to answer	9	15%	2	7.41%
Occupational status (χ^2=9.64, *df*=7, *p*=0.209)				
Occupational status, behavioral analyses (χ^2=10.39, *df*=7, *p*=0.167)				
Working full-time	9	15%	11	40.7%
Working part-time	7	11.7%	2	7.41%
Student full-time	9	15%	3	11.1%
Student part-time	3	5%	0	0%
Working & student	21	35%	9	33.3%
Homemaker	2	3.33%	0	0%
Retired	0	0%	0	0%
Volunteer worker	0	0%	0	0%
Seeking employment	6	10%	2	7.41%
Leave of absence	0	0%	0	0%
Disabled (other free response)	3	5%	0	0%
Relationship status (χ^2=2.94, *df*=3, *p*=0.400)				
Relationship status, behavioral analyses (χ^2=4.12, *df*=3, *p*=0.248)				
Single	48	80%	21	77.8%
Married	2	3.33%	3	11.1%
Divorced or separated	2	3.33%	0	0%
Widowed	0	0%	0	0%
Other	8	13.3%	3	11.1%
Alcohol frequency (χ^2=2.20, *df*=4, *p*=0.700)				
Alcohol frequency, behavioral analyses (χ^2=2.27, *df*=4, *p*=0.686)				
Never	15	25%	8	29.6%

	MDD		*Comparison*	
	**N**	**%**	**N**	**%**
Monthly or less	14	23.3%	7	25.9%
2-4 times a month	20	33.3%	5	18.5%
2-3 times a week	9	15%	6	22.2%
4 or more times a week	2	3.33%	1	3.7%
Alcohol amount (χ^2=2.46, *df*=4, *p*=0.651)				
Alcohol amount, behavioral analyses (χ^2=2.19, *df*=4, *p*=0.702)				
0 (N/A)	15	25%	8	29.6%
1 or 2	20	33.3%	10	37.0%
3 or 4	16	26.7%	5	18.5%
5 or 6	6	10.0%	4	14.8%
7, 8, or 9	3	5.0%	0	0%
10 or more	0	0%	0	0%
Caffeine amount (χ^2=2.59, *df*=4, *p*=0.623)				
Caffeine amount, behavioral analyses (χ^2=2.04, *df*=3, *p*=0.565)				
None	15	25%	7	25.9%
1 cup	29	48.3%	11	40.7%
2-3 cups	13	21.7%	9	33.3%
4-5 cups	2	3.33%	0	0%
6 or more cups	1	1.67%	0	0%
Tobacco (χ^2=2.71, *df*=1, *p*=0.099)				
Tobacco, behavioral analyses (χ^2=2.31, *df*=1, *p*=0.129)				
Uses tobacco	12	20%	1	3.7%
Does not use tobacco	48	80%	26	96.3%

	MDD		*Comparison*	
	**N**	**%**	**N**	**%**

Table S.I. 4: Demographic factors by diagnostic group. Column 1: demographic factor and response options. Continuous measures shown first with means (columns 2-3) and unpaired t-test statistics (columns 4-6, * indicates $p<0.05$). Years of education (self) was significantly different between groups, but not correlated with effort costs. Diagnostic groups were matched on all other variables, and this was also true within the subset of participants that were included in the task behavioral analyses (statistical tests denoted by 'behavioral analyses').

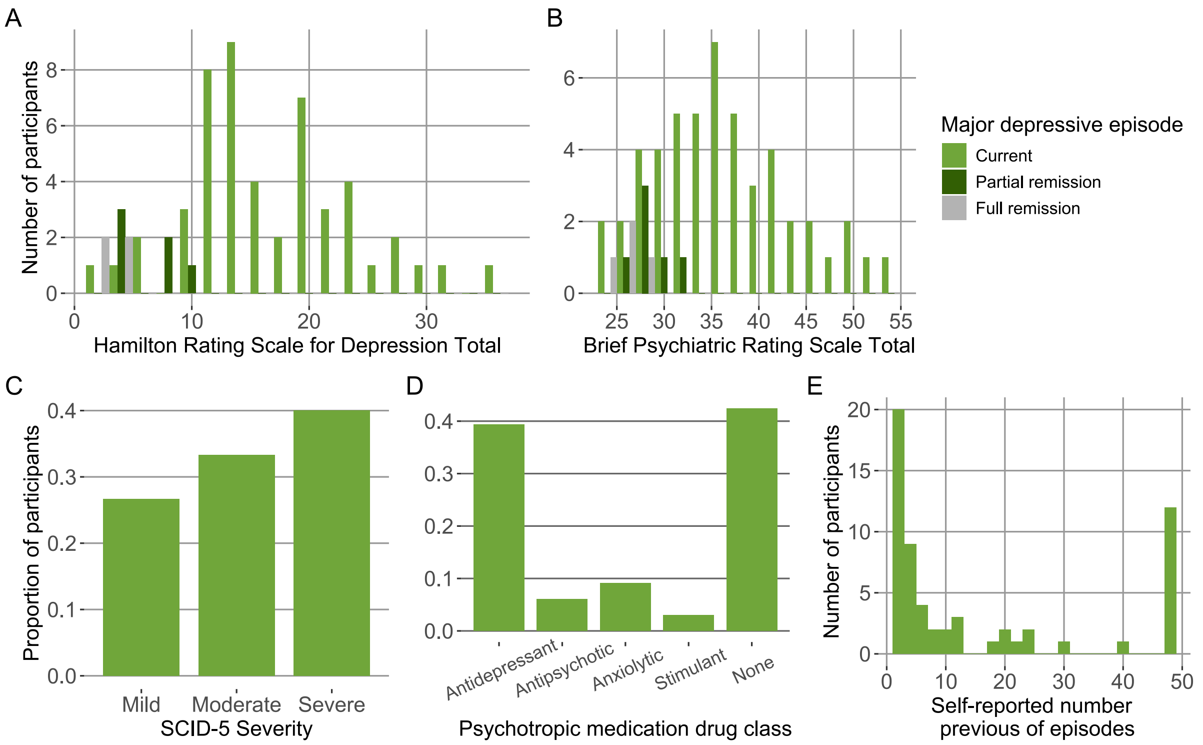

Figure S.I. 4: MDD sample characteristics. Histograms, A & B, y-axis: number of MDD participants, x-axis: A: Hamilton Depression Rating Scale total, B: Brief Psychiatric Rating Scale total, fill indicates major depressive episode status (light green indicates current, dark green indicates partial remission, and grey indicates full remission). C, D, & E, y-axis: proportion of MDD participants, C: Structured Clinical Interview for DSM-5 severity rating, D: psychotropic medication drug class, E: self-reported previous number of major depressive episodes.

14 Relationship between cognitive and physical effort costs

Previously, we found a significant positive correlation between cognitive and physical effort costs in a large online study (Experiment 1 (MSIT) of Bustamante et al. (2023), N=537, mean correlation=0.566, 95% HDI=0.355, 0.766). In a smaller undergraduate sample (Experiment 2 (N-Back) of Bustamante et al. (2023), N=81) there was no conclusive evidence for or against the correlation, as the highest density interval (HDI) was wide (mean correlation=0.048, 95% HDI=-0.369, 0.462). The present study uses the same N-Back version of the task as Experiment 2 of the original study with a comparable sample size (N=80 participants in total) and yielded a similarly wide posterior distribution (mean correlation=0.0532, 95% HDI=-0.240, 0.345, pd=0.365, Table S.I. 5). In both cases the credible interval overlapped with the posterior distribution from Experiment 1, and it may simply be the sample size is underpowered

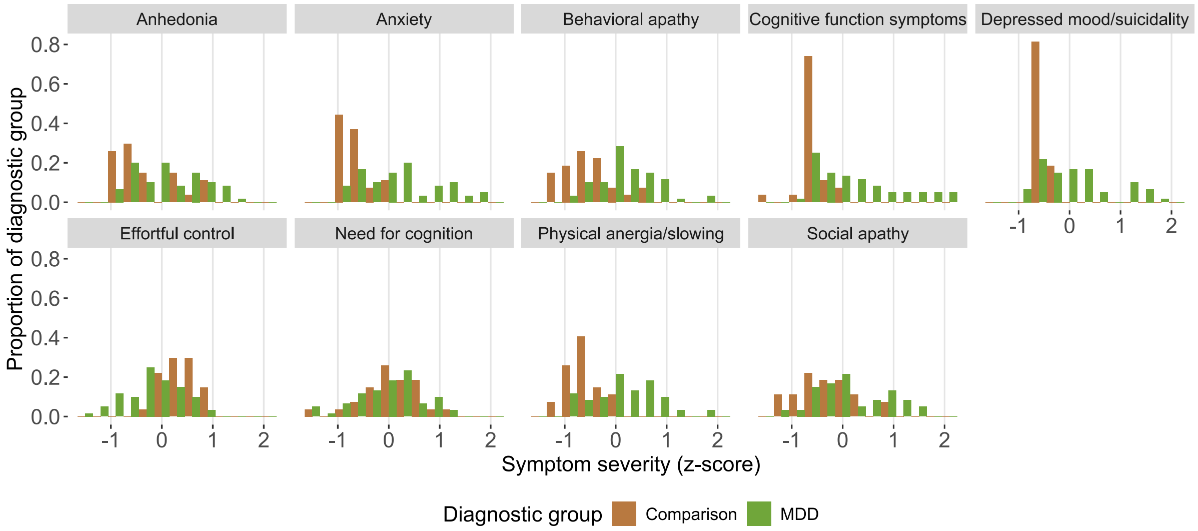

Figure S.I. 5: Symptom severity by domain and diagnostic group. Histogram, bar color indicates diagnostic group.

Parameter	Mean	Lower bound	Upper bound	*p*
Inverse Temperature (log)	-0.02	-0.20	0.15	0.408
Cognitive Low Effort Travel Cost	10.97	-6.69	27.19	0.098
Cognitive Effort Cost	11.71	7.10	16.47	<0.001
Physical Low Effort Travel Cost	18.18	0.00	35.06	0.025
Physical Effort Cost	8.59	3.03	14.21	0.001
Cognitive vs. Physical Effort Cost Correlation	0.05	-0.24	0.34	>0.365

Table S.I. 5: Group-level parameter posterior distribution values. Column 1: parameter, column 2: mean of the group-level posterior distribution, column 3: lower bound of 95% credible interval, column 4: upper bound of credible interval, column 5: Bayesian p-values.

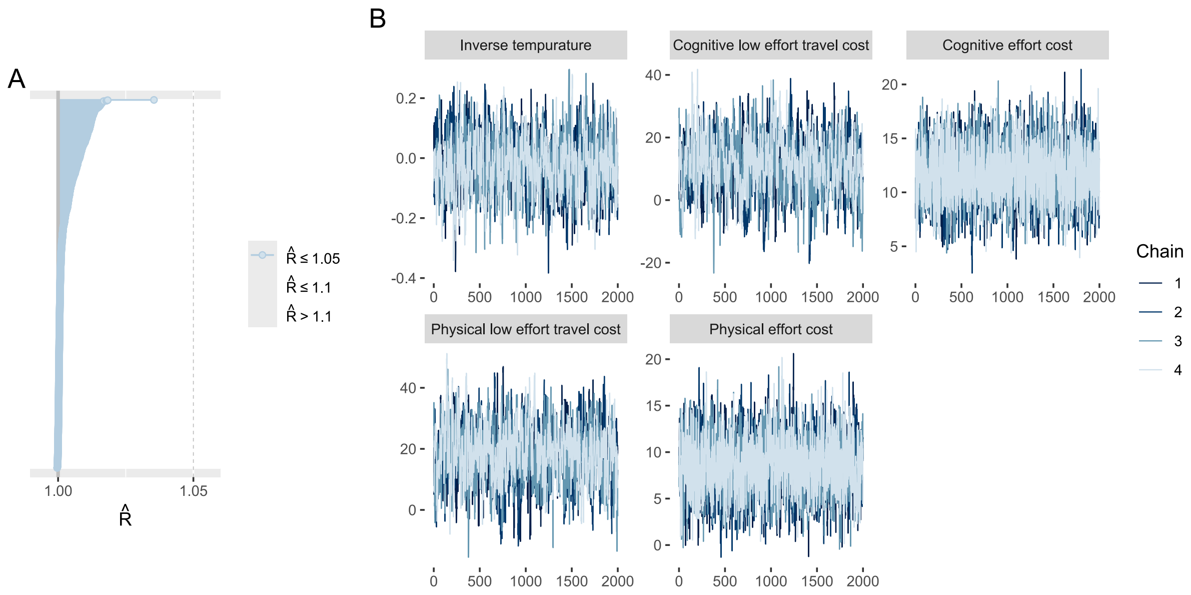

Figure S.I. 6: MVT model diagnostics. A: R̂ convergence diagnostic plot for all parameters, all of which are below the 1.05 cutoff (vertical dotted line) indicating model convergence. B: MCMC trace plots, color indicates chain number, overlapping traces suggests model convergence.

to detect the presence or absence of a correlation. Beyond this, there are several other differences between these task versions, MSIT involves interference control, whereas N-Back involves working memory. Furthermore, the N-Back version is longer in duration (56 versus 32 minutes of main task time), the longer travel time (8.33 seconds versus 20 seconds) requiring more sustained effort. More research is needed to understand under which conditions cognitive and physical effort-based decision making are connected versus dissociated.

Ours and previous research on the relationship between individual differences in cognitive and physical effort decision making have found moderate correlations (e.g., correlation=0.43 in Lopez-Gamundi & Wardle, correlation=0.35 in Tran et al., 2020). This unshared variance between the effort domains leaves open the possibility of decoupling of their relationship to specific psychiatric symptoms. Indeed in Experiment 1 of Bustamante et al. 2023 (using the MSIT to elicit cognitive effort), we found cognitive, but not physical effort cost loaded strongly onto the dimension predictive of symptoms in a CCA (Dimension 1). Here, we demonstrated differential relationships of cognitive and physical effort cost to symptoms by conducting a comparison of correlations (Meng, Rosenthal, and Rubin's z statistic, Meng, Rosenthal, and Rubin, 1992).

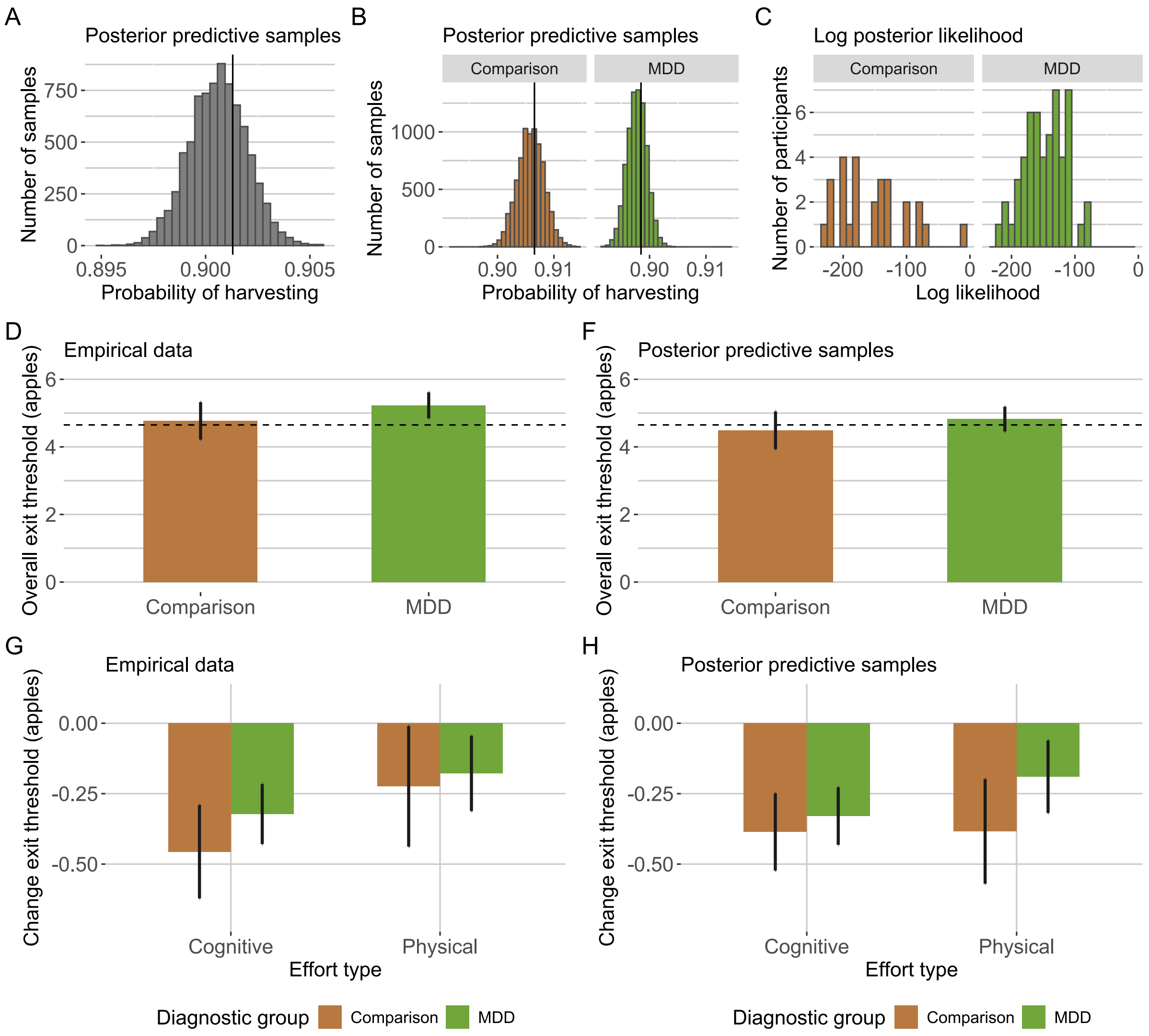

Figure S.I. 7: Posterior predictive check results by diagnostic group. A: x-axis, probability of choosing to harvest (1) or exit (0) across all trials, y-axis, number of MCMC samples, empirical observation indicated by vertical black line. B: x-axis, probability of choosing to harvest (1) or exit (0) across all trials for comparison participants (left) and MDD participants (right), y-axis, number of MCMC samples, empirical observations indicated by vertical black lines. C: x-axis, log posterior likelihood per participant for comparison (left) and MDD participants (right), y-axis number of participants. D: empirical data, x-axis, diagnostic group, y-axis, overall exit threshold (from low effort orchards), bars indicate group means, error bars indicate standard error of the mean, horizontal dotted line indicates best threshold from simulation. D: posterior predictive data, for each MCMC sample we computed the overall exit threshold per participant, and aggregated across samples to get the mean value per participant. Resulting plot shows group means and SEM matching C. E. empirical data, x-axis, effort type, y-axis, change exit threshold (high - low effort orchards), bars indicate group means, error bars indicate standard error of the mean, fill indicates diagnostic group. F. posterior predictive data, for each MCMC sample we computed the change in exit threshold per participant, and aggregated across samples to get the mean value per participant. Resulting plot shows group means and SEM matching E.

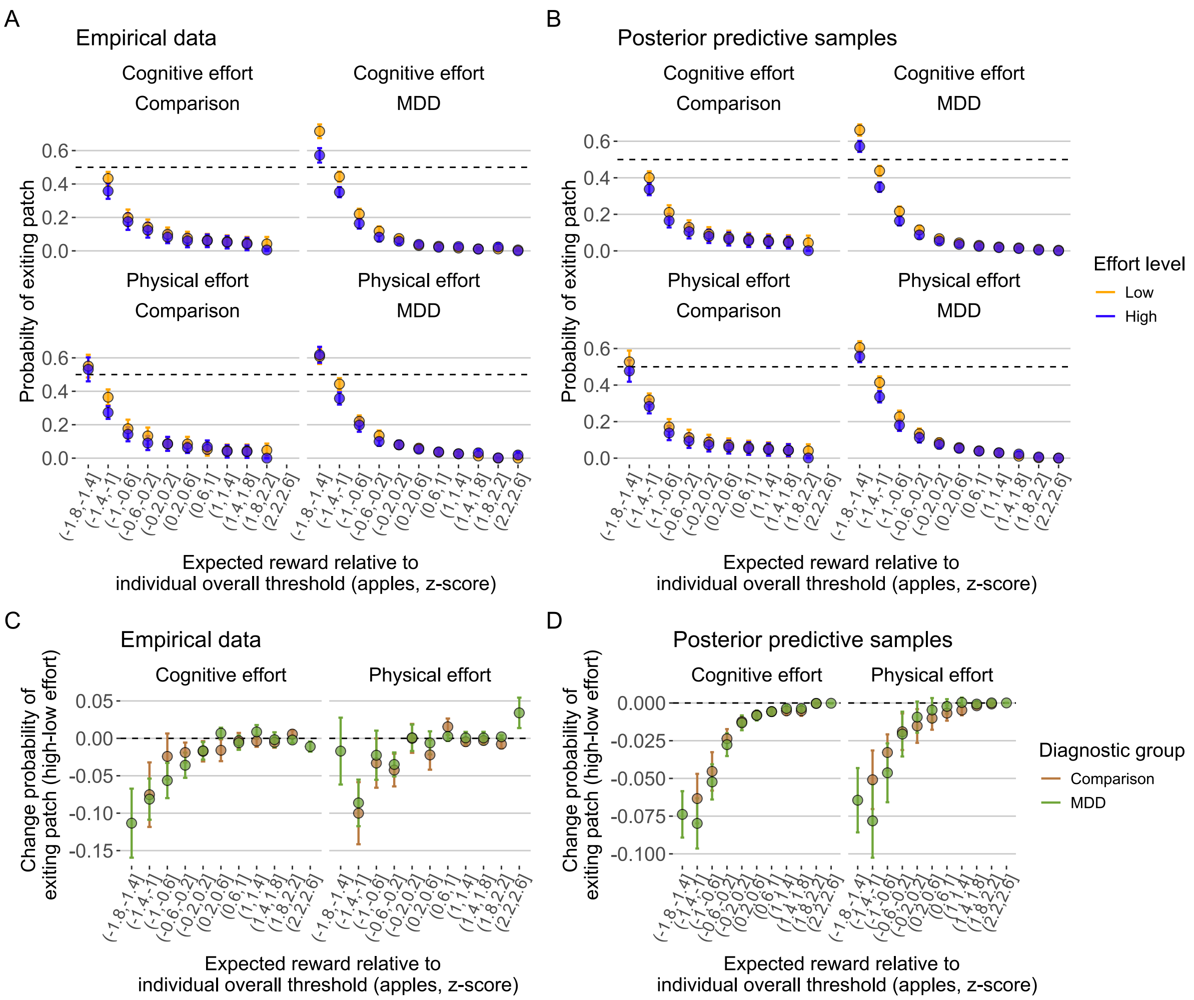

Figure S.I. 8: Probability of exiting by expected reward level, posterior predictive check results. A: empirical data, x-axis indicates relative expected reward, within each participant computed their overall exit threshold from the average of the low effort orchards using mixed-effects linear regression, then computed the difference between expected reward on each trial and the overall exit threshold (i.e., 'relative expected reward'), then z-scored this difference within participant and then binned the z-scores by 0.4 z-score units. y-axis indicates the probability of exiting the patch in each bin, points indicate group-level mean and error bars indicate standard error of the mean, points with fewer than 20 observations not displayed. First row indicates cognitive effort, and second row indicates physical effort, first column indicates comparison group, and second column indicates MDD group. B: posterior predictive data, same as panel A, except for each MCMC sample we computed the probability of exiting in each of the relative expected reward bins, and aggregated across samples to get the mean value per participant. Plot shows group-level means and SEM matching panel A. C. empirical data, x-axis, relative expected reward binned, y-axis, change in the probability of exiting patch (high - low effort orchards), bars indicate group-level means, error bars indicate standard error of the mean, fill indicates diagnostic group, brown color indicates comparison group, green color indicates MDD group, points with fewer than 25 observations not displayed. D. posterior predictive data, same as panel A, except for each MCMC sample we computed the change probability of exiting patch per participant, and aggregated across samples to get the mean value per participant. Resulting plot shows group means and SEM matching panel C.

15 Diagnostic group differences in effort-seeking

We used Pearson's Chi-squared tests with Yates' continuity correction to test whether the proportion of participants with negative effort costs differed by diagnostic group. For cognitive effort, 22.2% of comparison participants and 20% of MDD participants had a negative effort cost and there was no significant difference between groups (chi-square<0.001, df=1, p=1). For physical effort 29.6% of comparison participants and 28.3% of MDD participants had a negative effort cost and there was no significant difference between groups (chi-square=0.0, df=1, p=1).

16 Model-agnostic sensitivity to effort manipulation

The model-agnostic measure, change in exit threshold, showed that on average exit thresholds were lower in the cognitive and physical, high relative to low effort conditions (3-Back - 1-Back estimate=-0.092 log(apples), SE=0.022, df=74.24, t=-4.212, p<0.001; Larger - Smaller Number of Presses: -0.056 log(apples), SE=0.0249, df = 76.83, t-2.233, p<0.0285). There was no reliable interaction between diagnostic group and change in exit threshold for cognitive (t=0.991, df=73.78, p>0.325) nor physical effort (t=0.673, df=76.88, p>0.503). The MVT model group-level posterior parameters indicated high effort cost is greater than zero for both effort types (Table S.I. 5). There was considerable individual variation in willingness to exert effort, signaling differences in perceived effort costs (see Figure 1).

17 Fatigue effects group differences results

For both cognitive (t=-2.437, p<0.016) and physical effort (t=-2.617, p<0.010) we found a main effect of trial number within a round on model-agnostic exit thresholds, suggesting that overall thresholds may have been impacted by fatigue. However, there were no interactions with effort level, suggesting this process was not differentially affected in high or low effort orchards (cognitive effort, p>0.085, physical effort, p>0.024). Given that the effort costs depend on the difference between conditions, if they are comparably affected by fatigue, this

Symptom	Estimate	SE	*t*	*p*	$p_{adjusted}$
A. Cognitive effort cost, MDD, controlling for medication use					
Diagnostic group difference	-0.02	0.29	-0.08	0.935	
Overall depression	-0.39	0.16	-2.35	0.023*	
Anhedonia	-0.20	0.15	-1.32	0.192	0.227
Anxiety	-0.49	0.14	-3.62	0.001	0.005*
Behavioral apathy	-0.20	0.15	-1.32	0.195	0.227
Social apathy	0.01	0.15	0.06	0.953	0.953
Cognitive function symptoms	-0.30	0.14	-2.11	0.041	0.144
Depressed mood/suicidality	-0.26	0.17	-1.55	0.129	0.226
Physical anergia/slowing	-0.24	0.15	-1.59	0.118	0.226
B. Physical effort cost, MDD, controlling for medication use					
Diagnostic group difference	0.12	0.29	0.40	0.692	
Overall depression	0.04	0.17	0.22	0.828	
Anhedonia	0.42	0.14	2.90	0.006	0.035*
Anxiety	-0.04	0.16	-0.25	0.804	0.804
Behavioral apathy	0.40	0.15	2.68	0.010	0.035*
Social apathy	0.14	0.15	0.93	0.360	0.491
Cognitive function symptoms	0.27	0.16	1.65	0.106	0.247
Depressed mood/suicidality	0.14	0.17	0.81	0.421	0.491
Physical anergia/slowing	0.16	0.17	0.98	0.331	0.491
C. Overall exit threshold, MDD, controlling for medication use					
Diagnostic group difference	0.57	0.28	2.04	0.045*	
Psychotropic medication use	-0.568	0.274	-2.074	0.0415*	
Overall depression	-0.38	0.15	-2.47	0.017*	
Anhedonia	-0.29	0.14	-2.15	0.037	0.052
Anxiety	-0.33	0.14	-2.36	0.022	0.052
Behavioral apathy	-0.31	0.14	-2.22	0.031	0.052
Social apathy	-0.11	0.14	-0.82	0.414	0.41
Cognitive function symptoms	-0.22	0.14	-1.54	0.129	0.151
Depressed mood/suicidality	-0.40	0.15	-2.73	0.009	0.047*
Physical anergia/slowing	-0.35	0.14	-2.57	0.013	0.047*

Table S.I. 6: Symptoms effort cost regressions, controlling for psychotropic medication use, MDD participants. (A, MDD) Predict cognitive effort cost by overall depression severity and each symptom domain, controlling for cognitive task performance (3-Back D'), medication use, age, and years of education. (B, MDD) Predict physical effort cost by overall depression severity, and each symptom domain, controlling for physical task performance (% larger number of presses completed), BMI, medication use, age and years of education. (C) All participants, predict overall exit threshold (log apples, from low effort conditions) by diagnostic group (MDD-comparison) and psychotropic medication use. MDD participants, predict overall exit threshold (log apples, from low effort conditions) by symptom severity, medication use, age, and years of education (* indicates $p<0.05$, FDR correction within symptom models). All variables were scaled as input to the regressions.

**Diagnostic group effect parameter**	Mean	Lower	Upper	*pd*
Cognitive Effort Cost	-1.03	-8.05	6.08	0.39
Physical Effort Cost	-1.03	-8.05	6.08	0.39
Cognitive Low Effort Cost	1.81	-7.30	10.72	0.34
Physical Low Effort Cost	-3.06	-12.02	5.90	0.26
Inverse temperature (log)	0.10	-0.24	0.46	0.29
**Current depressed group effect parameter**	Mean	Lower	Upper	*p*
Cognitive Effort Cost	-1.39	-8.49	5.76	0.35
Physical Effort Cost	-1.73	-9.10	5.60	0.32
Cognitive Low Effort Cost	2.52	-6.53	11.77	0.29
Physical Low Effort Cost	-3.73	-13.01	5.40	0.21
Inverse temperature (log)	0.09	-0.29	0.49	0.32

Table S.I. 7: Diagnostic group difference MVT model. Group effect parameter for model that included all MDD participants. Current depressed group effect parameter for model that excluded participants in remission.

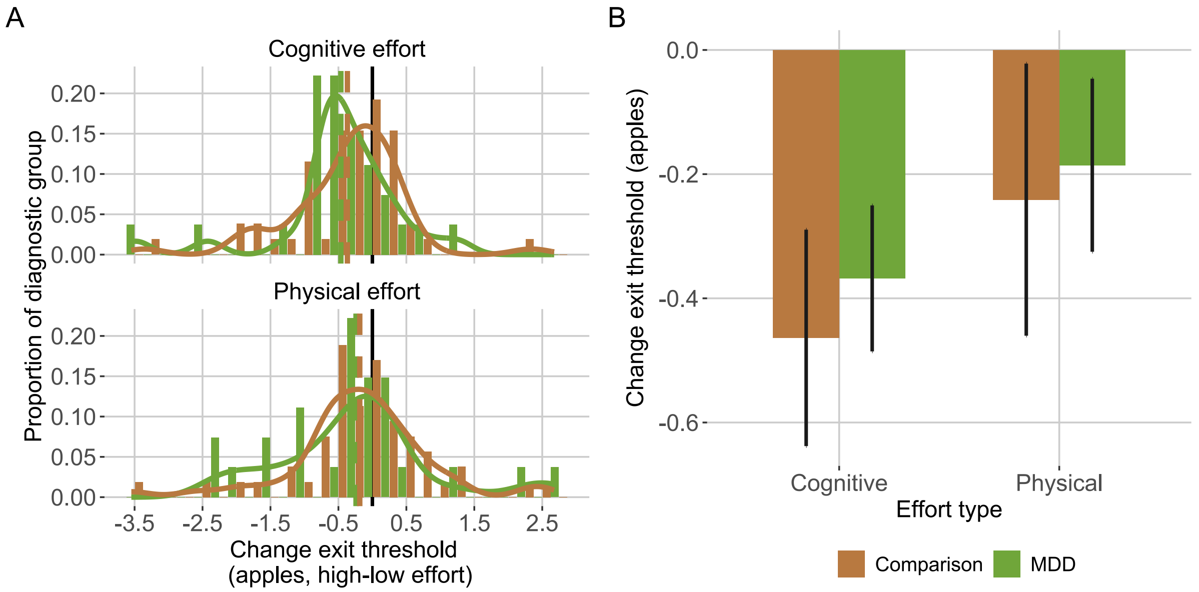

Figure S.I. 9: Model-agnostic change in exit threshold by diagnostic group. A: individual differences, error bars indicate 95% HDI, x-axis indicates cognitive effort condition, y-axis indicates physical effort condition. B: diagnostic group differences, x-axis indicates effort type and diagnostic group, y-axis indicates change in the exit threshold (high - low effort, apples).

would be unlikely to explain the effort cost specific effects. However, there is a limitation of this measure, which is that as participants get close to the end of a round they might suspect it is not worth traveling if the block will timeout before they get to the next orchard. This would be consistent with the effect of trial number observed, which could also be a combination of these factors.

Next we examined the three-way interaction between effort level, exit trial number, and diagnostic group. There were no diagnostic group differences in the effects of exit trial number (two-way interaction, cognitive effort, $p>0.901$, physical effort, $p>0.945$), nor in the three-way interaction (cognitive effort, $p>0.680$, physical effort, $p>0.105$). Therefore, fatigue or time in block related effects are unlikely to explain the lack of group-level differences in effort costs.

**Predicted variable**	**Estimate**	**SE**	***t***	***p***
Cognitive effort cost	-0.32	0.15	-2.13	0.038*
Physical effort cost	0.08	0.15	0.52	0.606
1-Back D'	-0.16	0.17	-0.92	0.361
3-Back D'	-0.12	0.18	-0.66	0.512
Smaller number of presses completed (%)	0.01	0.15	0.06	0.951
Larger number of presses completed (%)	-0.14	0.16	-0.91	0.369
Overall exit threshold	-0.44	0.14	-3.15	0.003*

Table S.I. 8: Self-reported overall depression regression results. Column 1: predicted variable, self-reported overall depression (PHQ-9) was used as a predictor variable in place of clinician-rated depression (HAMD). Results correspond to patterns identified using clinician rated depression. All variables were scaled as input to the regressions.

18 Additional task measures results

Across all the travel task measures tested we found few reliable diagnostic group differences (see Figure S.I. 11 and Table S.I. 11), including no difference in missed N-Back trials, cognitive task accuracy (D'), nor required keypresses determined in the calibration phase. We found the MDD group responded faster on average on the cognitive (N-Back) task (Figure S.I. 11). We found a significant effect of diagnostic group on percent of completed presses, in which the MDD group completed a larger percent of presses across conditions, but a significant diagnostic group by effort level interaction, in which the MDD group completed fewer

**Symptom**	**Estimate**	**SE**	***t***	***p***	$p_{adjusted}$
A. Cognitive effort cost, current MDD participants					
Overall depression	-0.29	0.21	-1.38	0.176	
Anhedonia	-0.13	0.17	-0.78	0.443	0.517
Anxiety	-0.50	0.15	-3.32	0.002	0.014*
Behavioral apathy	-0.21	0.16	-1.32	0.195	0.456
Social apathy	-0.07	0.17	-0.44	0.664	0.664
Cognitive function symptoms	-0.33	0.17	-1.99	0.054	0.189
Depressed mood/suicidality	-0.20	0.18	-1.11	0.273	0.477
Physical anergia/slowing	-0.17	0.18	-0.93	0.357	0.500
B. Physical effort cost, current MDD participants					
Overall depression	0.30	0.19	1.55	0.131	
Anhedonia	0.54	0.13	4.06	<0.001	<0.001*
Anxiety	0.06	0.16	0.35	0.729	0.851
Behavioral apathy	0.41	0.14	3.01	0.005	0.018*
Social apathy	0.02	0.15	0.14	0.888	0.888
Cognitive function symptoms	0.45	0.16	2.81	0.008	0.019*
Depressed mood/suicidality	0.23	0.16	1.43	0.163	0.228
Physical anergia/slowing	0.36	0.17	2.16	0.038	0.066
C. Cognitive effort cost, all participants					
Anhedonia	-0.13	0.12	-1.11	0.268	0.313
Anxiety	-0.40	0.12	-3.44	0.001	0.007*
Behavioral apathy	-0.21	0.12	-1.74	0.086	0.120
Social apathy	-0.02	0.12	-0.15	0.880	0.880
Cognitive function symptoms	-0.24	0.12	-2.02	0.047	0.120
Depressed mood/suicidality	-0.23	0.13	-1.75	0.085	0.120
Physical anergia/slowing	-0.21	0.12	-1.75	0.085	0.120
D. Physical effort cost, all participants					
Anhedonia	0.27	0.12	2.30	0.024	0.168
Anxiety	-0.04	0.13	-0.32	0.753	0.835
Behavioral apathy	0.21	0.12	1.71	0.091	0.318
Social apathy	0.03	0.12	0.21	0.835	0.835
Cognitive function symptoms	0.14	0.12	1.19	0.239	0.558
Depressed mood/suicidality	0.10	0.14	0.74	0.459	0.803
Physical anergia/slowing	0.06	0.13	0.45	0.654	0.835

Table S.I. 9: Symptoms effort cost regressions, current MDD only, and all participants. (A, current MDD, C, all participants) Predict cognitive effort cost by overall depression severity and each symptom domain, controlling for cognitive task performance (3-Back D'), age, and years of education. (B, current MDD, D, all participants) Predict physical effort cost by overall depression severity, and each symptom domain, controlling for physical task performance (% larger number of presses completed), BMI, age and years of education (* indicates $p<0.05$, FDR correction within symptom models). All variables were scaled as input to the regressions.

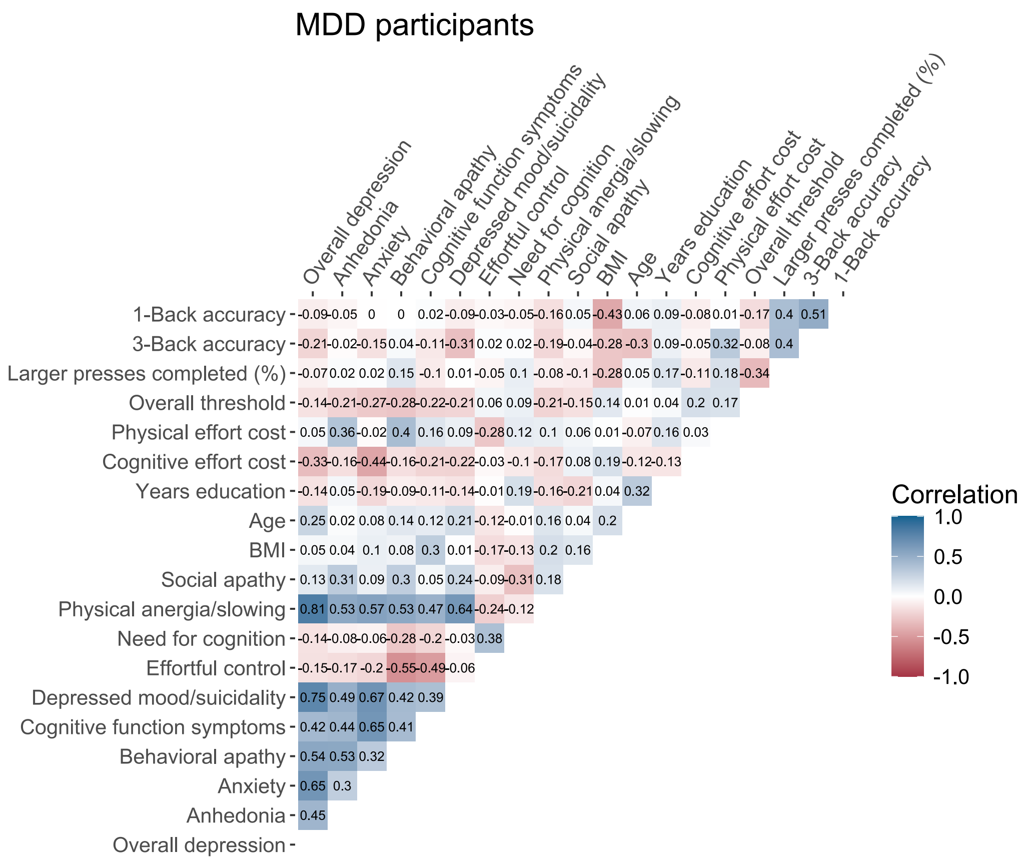

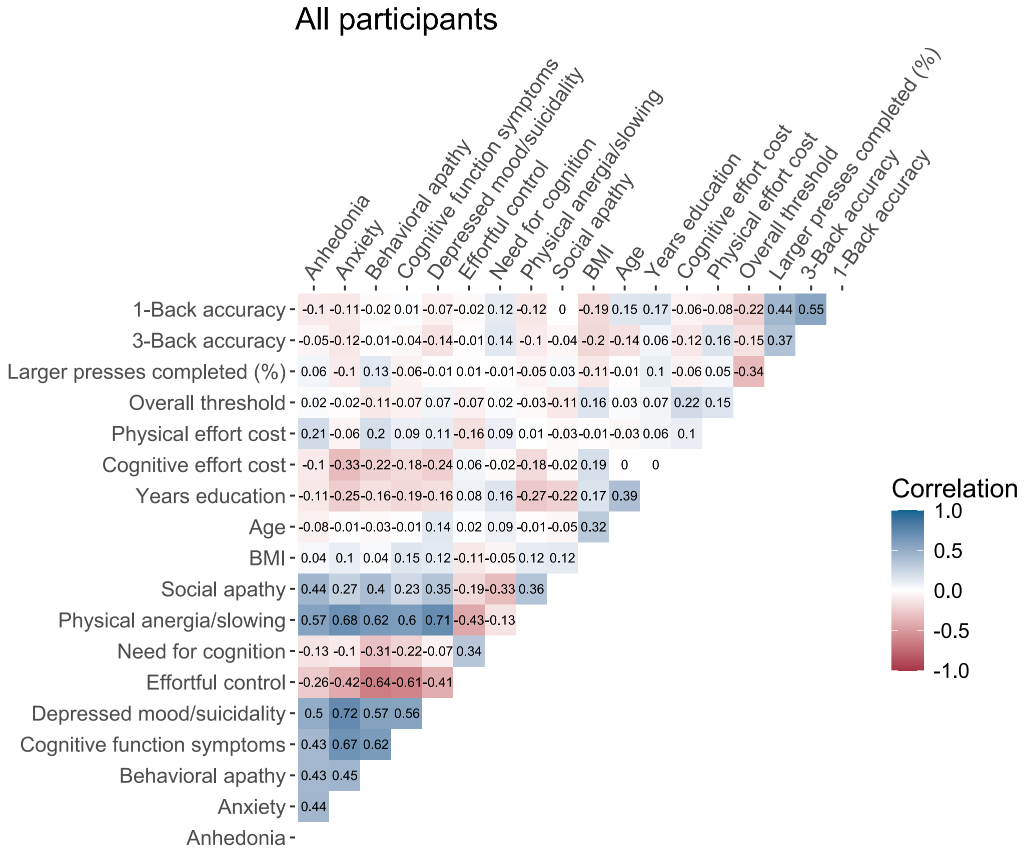

Figure S.I. 10: Task behavior symptom heatmap. Top MDD group only, bottom all participants (spearman correlation matrix).

**Symptom**	**Estimate**	**SE**	***t***	***p***	$p_{adjusted}$
Overall depression	0.033	0.161	0.207	0.837	
Anhedonia	0.270	0.137	1.970	0.055	0.382
Anxiety	0.041	0.146	0.280	0.781	0.972
Behavioral apathy	0.234	0.145	1.620	0.112	0.391
Social apathy	0.012	0.140	0.087	0.931	0.972
Cognitive function symptoms	-0.005	0.145	-0.035	0.972	0.972
Depressed mood/suicidality	0.210	0.154	1.360	0.180	0.420
Physical anergia/slowing	-0.085	0.146	-0.585	0.561	0.972

Table S.I. 10: Symptoms inverse temperature regressions, MDD only. Predict inverse temperature by overall depression severity and each symptom domain, controlling for age, and years of education (* indicates $p<0.05$, FDR correction within symptom models). All variables were scaled as input to the regressions.

presses. To decompose this effect, we ran the same regression separately for each effort level. There was no reliable group effect on the percent of smaller number of presses completed, but the MDD group completed a lower percent of required keypresses in the larger press condition.

We found that cognitive and physical effort costs were dissociable from task performance in the Effort Foraging Task, this may suggest a disconnect between effort selection and effort execution (as suggested in, O'Reilly, Hazy, Mollick, Mackie, and Herd, 2014). There was no reliable association between cognitive effort cost and cognitive task performance (Figure S.I. 12, model 1: 3-Back D', $p>0.47$, 1-Back D', $p>0.85$, all participants, model 2: change D', $p>0.75$). Likewise, there was no reliable relationship between physical effort cost and the percent of key presses completed (model 3: larger number of presses, $p>0.83$, smaller number of presses, $p>0.31$, consistent with that was found in Culbreth et al., 2023).

Neither cognitive nor physical performance was reliably related to overall depression (predict Hamilton Depression Rating Scale Total controlling for age by 3-Back D', $p>0.16$, 1-Back D', $p>0.86$). While anxiety symptoms were associated with cognitive effort costs, they were not associated with cognitive task performance (MDD group predict by anxiety symptoms controlling for age by 3-Back D', $p>0.21$, 1-Back D', $p>0.79$). On the other hand, anhedonia symptoms were related to a lower percentage of completed keypresses in the low ($t=2.68$, $p<0.010$), but not high ($p>0.311$) physical effort condition.

Overall depression was not related to cognitive task performance (Figure S.I. 12, predict

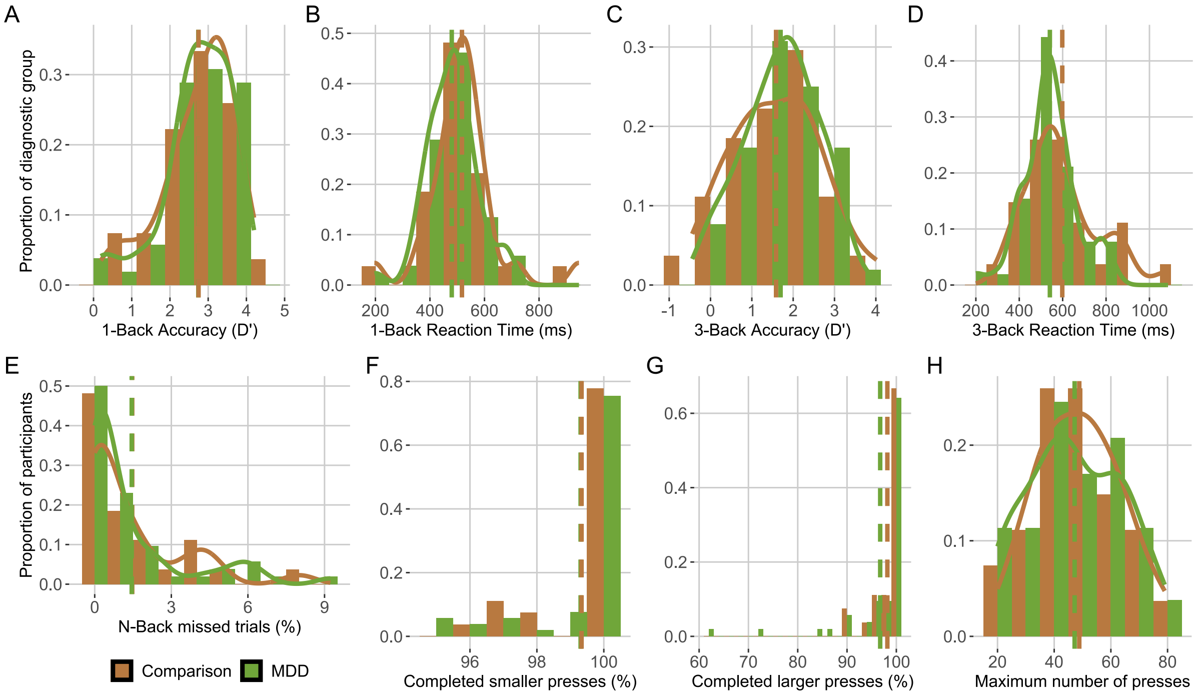

Figure S.I. 11: Diagnostic group difference in travel task performance. A: 1-Back accuracy (D'), B: 1-Back Reaction Time (ms), C: 3-Back accuracy (D'), D: 3-Back Reaction Time (ms), E: percent of N-Back trials missed responding before the RT deadline, F: Physical low effort performance (percent completed smaller number of presses), G: Physical high effort performance (percent completed larger number of presses), H: Maximum number of presses determined in a calibration phase. There were no diagnostic group differences except that the MDD group responded faster on average on the cognitive (N-Back) task (A & C), and completed fewer presses in the high effort physical condition (G).

**Predicted variable**	**Predictor variable**	**Estimate**	**SE**	**df**	***t***	***p***
N-Back missed trials (%)	Group	-0.021	0.495	77	-0.042	0.966
N-Back RT (log)	Group	-0.163	0.123	78.63	-1.35	0.181
N-Back RT (log)	Group*correct*N-Back	-0.197	0.084	4955	-2.335	0.020
N-Back RT (log)	Group*correct* target	-0.273	0.009	2450	-3.206	0.001
Accuracy (D')	Group	-0.008	0.222	113.6	-0.034	0.973
Accuracy (D')	Group*N-Back level	0.117	0.202	77	0.577	0.566
Required keypresses	Group	-2.00	3.640	77	-0.550	0.584
Completed presses (%)	Group	0.272	0.970	101	0.281	0.780
Completed presses (%)	Group*effort level	-2.379	0.675	3237.4	-3.524	0.0004
Completed larger presses	Group	-1.490	1.510	79.33	-0.987	0.327
Completed smaller presses	Group	-0.010	0.358	1650	-0.027	0.978

Table S.I. 11: Travel task performance diagnostic group differences. Column 1: predicted variable in regression, column 2: predictor variable, column 3: regression estimate, column 4: standard error (SE), column 5: degrees of freedom, column 6: t-statistic, column 7: p-value.

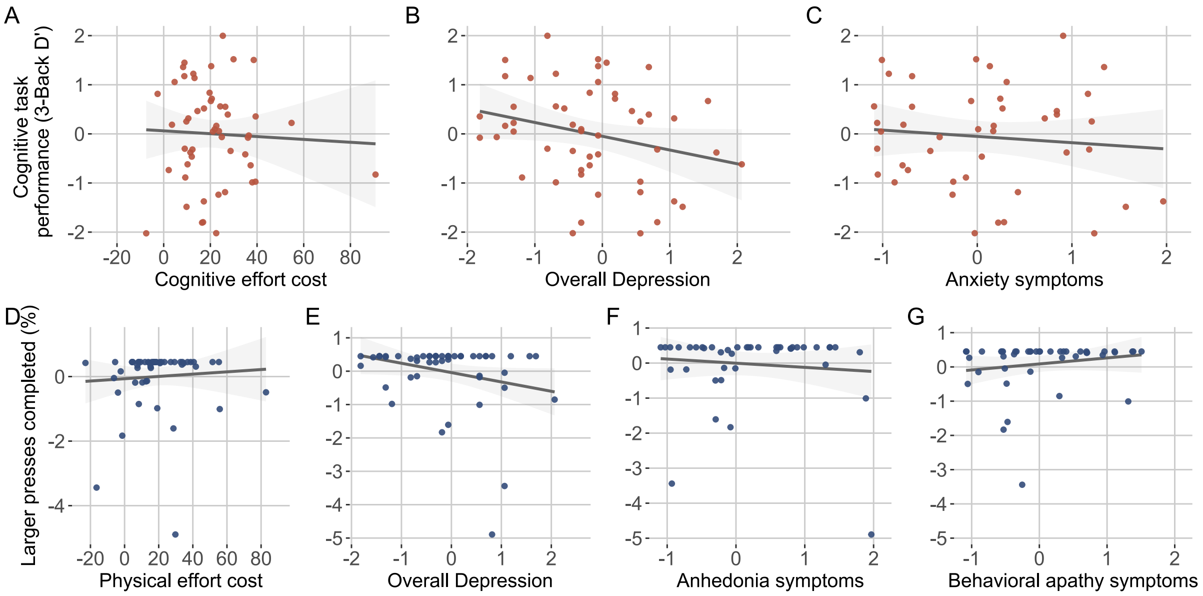

Figure S.I. 12: Travel task performance relationship to effort costs, overall depression, anhedonia, and anxiety. Only MDD group. Cognitive travel task performance (3-Back Accuracy (D'), y-axis) not reliably related to, A: cognitive effort cost (p>0.470), B: overall depression (HAMD, z-score, p>0.16), nor C: anxiety symptoms (x-axis, z-score, p>0.21). Physical travel task performance (percent of larger number of presses completed, y-axis) not reliably related to, D: physical effort cost (p>0.828), E: overall depression (p>0.073), nor F: anhedonia symptoms (x-axis, p>0.318), nor G: behavioral apathy symptoms (p>0.240).

HAMD total controlling for age by 3-Back D', p>0.16, 1-Back D', p>0.86) nor for physical task performance (predict HAMD total controlling for age and BMI by Larger number of presses, p>0.073, Smaller number of presses, p>0.370). While anxiety symptoms were associated with cognitive effort costs, they were not associated with cognitive task performance (MDD group predict by anxiety symptoms by 3-Back D', controlling for age, p>0.21, and 1-Back D', p>0.79). Anhedonia symptoms were related to the percent of smaller number of presses completed (t=2.70, p<0.010) but not to the percent of larger number of presses (p>0.318). Behavioral apathy symptoms were related to the percent of smaller number of presses completed (p>0.200) but not to the percent of larger number of presses (p>0.240).

**Symptom**	**Estimate**	**SE**	***t***	***p***	***p*** $_{adjusted}$
A. Overall exit threshold, current MDD					
Overall depression	-0.42	0.20	-2.12	0.040*	
Anhedonia	-0.24	0.16	-1.49	0.145	0.203
Anxiety	-0.31	0.16	-1.93	0.061	0.147
Behavioral apathy	-0.28	0.16	-1.77	0.084	0.147
Social apathy	-0.20	0.16	-1.25	0.219	0.255
Cognitive function symptoms	-0.16	0.17	-0.93	0.356	0.356
Depressed mood/suicidality	-0.44	0.16	-2.66	0.011*	0.079
Physical anergia/slowing	-0.33	0.17	-1.90	0.065	0.147
B. Overall exit threshold, all participants					
Anhedonia	-0.03	0.12	-0.29	0.775	0.775
Anxiety	-0.13	0.12	-1.07	0.289	0.506
Behavioral apathy	-0.14	0.12	-1.17	0.247	0.506
Social apathy	-0.05	0.12	-0.41	0.687	0.775
Cognitive function symptoms	-0.10	0.12	-0.86	0.395	0.553
Depressed mood/suicidality	-0.22	0.13	-1.65	0.104	0.506
Physical anergia/slowing	-0.13	0.12	-1.08	0.285	0.506

Table S.I. 12: Overall exit threshold relationship to symptoms (current MDD group, and all participants). Predicting individual differences in overall exit thresholds (log, from low effort conditions) by overall depression severity, and each symptom domain, controlling for age and years of education (* indicates $p<0.05$, FDR correction within symptom models). All variables were scaled as input to the regressions.

Supplementary References

Ang, Y.-S., Lockwood, P., Apps, M. A. J., Muhammed, K., & Husain, M. (2017). Distinct subtypes of apathy revealed by the apathy motivation index. *PloS One*, *12*(1), e0169938. doi:10.1371/journal.pone.0169938

Bustamante, L. A., Oshinowo, T., Lee, J. R., Tong, E., Burton, A. R., Shenhav, A., ... Daw, N. D. (2023). Effort foraging task reveals positive correlation between individual differences in the cost of cognitive and physical effort in humans. *Proceedings of the National Academy of Sciences*, *120*(50), e2221510120. Publisher: Proceedings of the National Academy of Sciences. doi:10.1073/pnas.2221510120

Cacioppo, J. T., Petty, R. E., & Kao, C. F. (1984). The efficient assessment of need for cognition. *Journal of Personality Assessment*, *48*(3), 306–307. doi:10.1207/s15327752jpa4803_13

Champely, S., Ekstrom, C., Dalgaard, P., Gill, J., Weibelzahl, S., Anandkumar, A., ... Rosario, H. D. (2020, March 17). Pwr: Basic functions for power analysis (Version 1.3-0). Retrieved June 9, 2024, from https://cran.r-project.org/web/packages/pwr/index.html

Cohen, J. (1992). A power primer. *Psychological Bulletin*, *112*(1), 155–159. doi:10.1037/0033-2909.112.1.155

Cohen, J. D., Forman, S. D., Braver, T. S., Casey, B. J., Servan-Schreiber, D., & Noll, D. C. (1994). Activation of the prefrontal cortex in a nonspatial working memory task with functional MRI. *Human Brain Mapping*, *1*(4), 293–304. doi:10.1002/hbm.460010407

Culbreth, A. J., Moran, E. K., Mahaphanit, W., Erickson, M. A., Boudewyn, M. A., Frank, M. J., ... Gold, J. M. (2023). A transdiagnostic study of effort-cost decision-making in psychotic and mood disorders. *Schizophrenia Bulletin*, sbad155. doi:10.1093/schbul/sbad155

Evans, D. E., & Rothbart, M. K. (2007). Developing a model for adult temperament. *Journal of Research in Personality*, *41*(4), 868–888. doi:10.1016/j.jrp.2006.11.002

Fava, M., Iosifescu, D. V., Pedrelli, P., & Baer, L. (2009). Reliability and validity of the massachusetts general hospital cognitive and physical functioning questionnaire. *Psychotherapy and Psychosomatics*, *78*(2), 91–97. doi:10.1159/000201934

First, M. B. (2015). Structured clinical interview for the DSM (SCID). In *The encyclopedia of clinical psychology* (pp. 1–6). _eprint: https://onlinelibrary.wiley.com/doi/pdf/10.1002/9781118625392. doi:10.1002/9781118625392.wbecp351

Gabry, J., Mahr, T., Bürkner, P.-C., Modrák, M., Barrett, M., Weber, F., … Vehtari, A. (2024, February 15). Bayesplot: Plotting for bayesian models (Version 1.11.1). Retrieved June 6, 2024, from https://cran.r-project.org/web/packages/bayesplot/index.html

Hamilton, M. (1960). A rating scale for depression. *Journal of Neurology, Neurosurgery, and Psychiatry*, *23*, 56–62. doi:10.1136/jnnp.23.1.56

Kroenke, K., Spitzer, R. L., & Williams, J. B. (2001). The PHQ-9: Validity of a brief depression severity measure. *Journal of General Internal Medicine*, *16*(9), 606–613. doi:10.1046/j.1525-1497.2001.016009606.x

Lewandowski, D., Kurowicka, D., & Joe, H. (2009). Generating random correlation matrices based on vines and extended onion method. *Journal of Multivariate Analysis*, *100*(9), 1989–2001. doi:10.1016/j.jmva.2009.04.008

Meng, X.-l., Rosenthal, R., & Rubin, D. B. (1992). Comparing correlated correlation coefficients. *Psychological Bulletin*, *111*, 172–175. Place: US Publisher: American Psychological Association. doi:10.1037/0033-2909.111.1.172

Nakonezny, P. A., Carmody, T. J., Morris, D. W., Kurian, B. T., & Trivedi, M. H. (2010). Psychometric evaluation of the snaith-hamilton pleasure scale in adult outpatients with major depressive disorder. *International Clinical Psychopharmacology*, *25*(6), 328–333. doi:10.1097/YIC.0b013e32833eb5ee

O'Reilly, R. C., Hazy, T. E., Mollick, J., Mackie, P., & Herd, S. (2014, April 30). Goal-driven cognition in the brain: A computational framework. doi:10.48550/arXiv.1404.7591. arXiv: 1404.7591[q-bio]

Overall, J. E., & Gorham, D. R. (1962). The brief psychiatric rating scale. *Psychological Reports*, *10*(3), 799–812. Publisher: SAGE Publications Inc. doi:10.2466/pr0.1962.10.3.799

RCoreTeam. (2015). R: A language and environment for statistical computing. R Foundation for Statistical Computing, Vienna, Austria. Retrieved February 13, 2022, from https://www.r-project.org/

Spitzer, R. L., Kroenke, K., Williams, J. B. W., & Löwe, B. (2006). A brief measure for assessing generalized anxiety disorder: The GAD-7. *Archives of Internal Medicine*, *166*(10), 1092–1097. doi:10.1001/archinte.166.10.1092

Stan. (2021). CmdStanR: The r interface to CmdStan — cmdstanr-package. Retrieved November 2, 2022, from https://mc-stan.org/cmdstanr/reference/cmdstanr-package.html

Team, S. D. (2021). Stan modeling language users guide and reference manual (Version 2.29). Retrieved February 20, 2022, from https://mc-stan.org

Wechsler, D. (2001). Wechsler test of adult reading: WTAR. *Psychological Corporation*.
